# Comparison of the Effects of Sorafenib and Stem Cell Secretome on HepG2 Cancer Cells with Respect to the Ras/Raf/MEK/ERK Pathway

**DOI:** 10.3390/jcm15176738

**Published:** 2026-08-30

**Authors:** Aleksandra Gładyś, Aleksandra Skubis-Sikora, Bartosz Sikora, Kinga Pogoda-Mieszczak, Patrycja Wieczorek, Edyta Bogunia, Piotr Czekaj

**Affiliations:** Department of Cytophysiology, Chair of Histology and Embryology, Faculty of Medical Sciences in Katowice, Medical University of Silesia in Katowice, Medyków 18, 40-752 Katowice, Poland; d201163@365.sum.edu.pl (A.G.); askubis@sum.edu.pl (A.S.-S.); bsikora@sum.edu.pl (B.S.); kpogoda-mieszczak@sum.edu.pl (K.P.-M.); pszmytkowska@sum.edu.pl (P.W.); edytab@sum.edu.pl (E.B.)

**Keywords:** adipose tissue-derived stem cells, amniotic membrane-derived cells, sorafenib, Ras/Raf/MEK/ERK pathway, secretome, conditioned medium

## Abstract

**Background/Objective:** Liver cancer is a global health challenge due to its resistance to most systemic therapies. Human mesenchymal and epithelial stem cells exhibit anti-proliferative and pro-apoptotic effects on some cancer cell lines, which can be used to support standard therapy. The aim of this study was to investigate the effects of conditioned media (CM) derived from mesenchymal adipose tissue-derived stem cells (hADSCs) and amniotic membrane-derived cells expressing both mesenchymal and epithelial markers (hACs) on HepG2 liver cancer cells in vitro and to identify possible similarities to the action of sorafenib, a standard drug in hepatocellular carcinoma (HCC) therapy. **Methods:** HepG2 cells were cultured with hADSC-derived CM (CM-hADSC) or hAC-derived CM (CM-hAC), either alone or with 7.5 μm sorafenib for 48 h. HepG2 cell viability and the expression of genes and/or proteins related to the apoptosis, cell-cycle, and Ras/Raf/MEK/ERK signaling pathway, were assessed. **Results:** The effect of sorafenib administration alone, consisting in reducing HepG2 cell viability, cell cycle inhibition, and reducing the expression of the alpha-fetoprotein (*AFP*) gene and most proteins related to the Ras/Raf/MEK/ERK pathway, was dominant over the effect of CM administered in combination with one of them. The effect of sorafenib on increasing the number of early apoptotic cells was not associated with increased Bax protein expression or with an increased proportion of cleaved forms of caspase-7 and caspase-9. The effect of CM-hAC was often different from that of CM-hADSC and consisted in enhancing the inhibitory effect of sorafenib at the cell cycle level and increasing the number of apoptotic cells and caspase mRNA expression, while activating the Ras/Raf/MEK/ERK signaling pathway and inducing *AFP* gene expression. **Conclusions:** Combined administration of sorafenib and some CM may produce the desired effects by enhancing the action of the drug. However, some of the effects of sorafenib and CM derived from different stem cell types were significantly different, which means that the role of stem cell secretome as a potential anticancer therapy requires further investigation, taking into account different types of cancer cell lines and a thorough analysis of the secretome composition.

## 1. Introduction

According to 2022 data, lung, colon, and liver cancer are the three leading causes of cancer deaths [1]. Hepatocellular carcinoma (HCC) is the most common type of liver cancer [2]. Alterations in the mitogen-activated protein kinase (MAPK) pathway, comprising the Ras/Raf/MAPK (MEK)—extracellular-signal-regulated kinase (ERK) cascade, play a crucial role in the occurrence and development of HCC [3]. Mutations and overexpression of genes in the Ras/Raf/MEK/ERK pathway are observed in HCC [4,5]. Therefore, inhibition of the Ras/Raf/MEK/ERK pathway is considered a potential mechanism of action for novel anticancer drugs [6]. Moreover, studies have indicated a possible association between Ras/Raf/MEK/ERK overexpression and poorer overall survival (OS) in HCC [7]. Serum alpha-fetoprotein (AFP), a protein produced by hepatocellular carcinoma cells, is used to monitor HCC patients [8].

According to the international guidelines [9,10], standard treatment depends on disease stage and includes surgical resection and targeted therapies. For patients with well-preserved liver function and early or intermediate HCC, treatment options include liver resection, orthotopic liver transplantation, radiotherapy or embolization. Despite the fact that detailed treatment schemes are subject to variation on a country-by-country basis, in advanced, unresectable HCC, standard treatment includes immunotherapy or oral tyrosine kinase inhibitors such as sorafenib, regorafenib and lenvatinib, with immunotherapy agents as the first-line options [10].

Sorafenib targets Raf-1 kinase, thereby inhibiting the Ras/Raf/MEK/ERK pathway in HCC cells [6,11]. Notably, sorafenib reduces MEK and ERK phosphorylation, key components of the Ras/Raf/MEK/ERK pathway [11], and downregulates the expression of cyclin D1 and E1 [11,12]. As a result, sorafenib suppresses cell proliferation and promotes apoptosis in HCC cells [11]. Besides inhibiting Raf kinases, sorafenib also targets vascular endothelial growth factor receptors (VEGFRs), and platelet-derived growth factor receptor beta (PDGFR-β), thereby suppressing tumor growth and angiogenesis [13,14]. Clinically, HCC patients treated with sorafenib have improved prognosis, as the drug prolongs time to progression and overall survival [6].

Because standard therapies for HCC remain limited in effectiveness, novel treatment opportunities are being investigated. The use of stem cells in oncology has recently gained attention owing to their secretion of soluble factors with potential anti-proliferative and pro-apoptotic effects on cancer cells. Among them are mesenchymal stem cells (MSCs), such as human adipose-derived stem cells (hADSCs) [15] and human amniotic mesenchymal stem cells (hAM-MSCs) [16], as well as human amniotic epithelial cells (hAECs) [17] which can be readily isolated from human tissues without ethical or legal concerns [16,18].

The therapeutic potential of hADSCs has been investigated in soft-tissue regeneration and reconstruction, as well as in other conditions such as osteoarthritis, diabetes, heart failure and MSC-loaded scaffolds for bone regeneration [19,20]. Other pre-clinical studies have demonstrated the potential use of hADSCs in liver regeneration, particularly in acute liver injury [21], chronic fibrosis [22,23,24,25], non-alcoholic fatty liver disease (NAFLD) [26], and in a liver transplantation model [27], mainly through the alleviation of acute and chronic tissue damage and the suppression of transplant rejection. In some pre-clinical studies, hADSCs have displayed anticancer effects against various cancer types [28,29], including HCC [30]. In HCC, both hADSCs and hADSC-derived secretome have been shown to inhibit proliferation [31,32], induce apoptosis [30,32,33], and reduce tumor growth [30]. However, observations that indicate the activity of the ADSCs secretome supporting the growth of cancer cells should also be taken into account [15].

Amnion-derived cells are known for their phenotypic diversity, including variability in cell marker expression. These include hAM-MSCs and hAECs [34]. hAM-MSCs have been shown to exhibit differentiation potential towards adipogenic, osteogenic, and chondrogenic lineages, as well as myocyte-, hepatocyte-, and neuron-like cells [35]. Their regenerative potential has been demonstrated in pre-clinical studies of bone [36], cartilage [37], skin [38], endometrial [39], and cardiac [40] repair. Similarly to hADSCs, hAM-MSCs can support liver regeneration in models of acute injury [41] and fibrosis [42]. Anticancer effects have also been observed in HCC [43] and other cancer types [44,45,46]. However, hAM-MSCs can differ phenotypically from other mesenchymal cells, such as hADSCs [47].

hAECs may differentiate or undergo epithelial–mesenchymal transition (EMT) in vitro [48]. Cells resulting from EMT, although exhibiting typical mesenchymal markers, can also partially express epithelial markers such as cytokeratins [49]. This population maintains an intermediate phenotype with predominantly mesenchymal and partially epithelial features. Thus, the secretomes of amniotic epithelial and mesenchymal stem cells can differ, as do their paracrine effects.

We hypothesized that the secretomes of hADSCs and amnion-derived cells expressing epithelial and mesenchymal markers (hACs) may act therapeutically and complementarily with sorafenib in HCC cells. The aim of the study was to compare the effect of conditioned media (CM) derived from hADSC and hAC cultures and sorafenib on HepG2 cells, with particular emphasis on the expression of genes and proteins of the Ras/Raf/MEK/ERK signaling pathway.

## 2. Materials and Methods

### 2.1. Study Design

**Figure 1:**
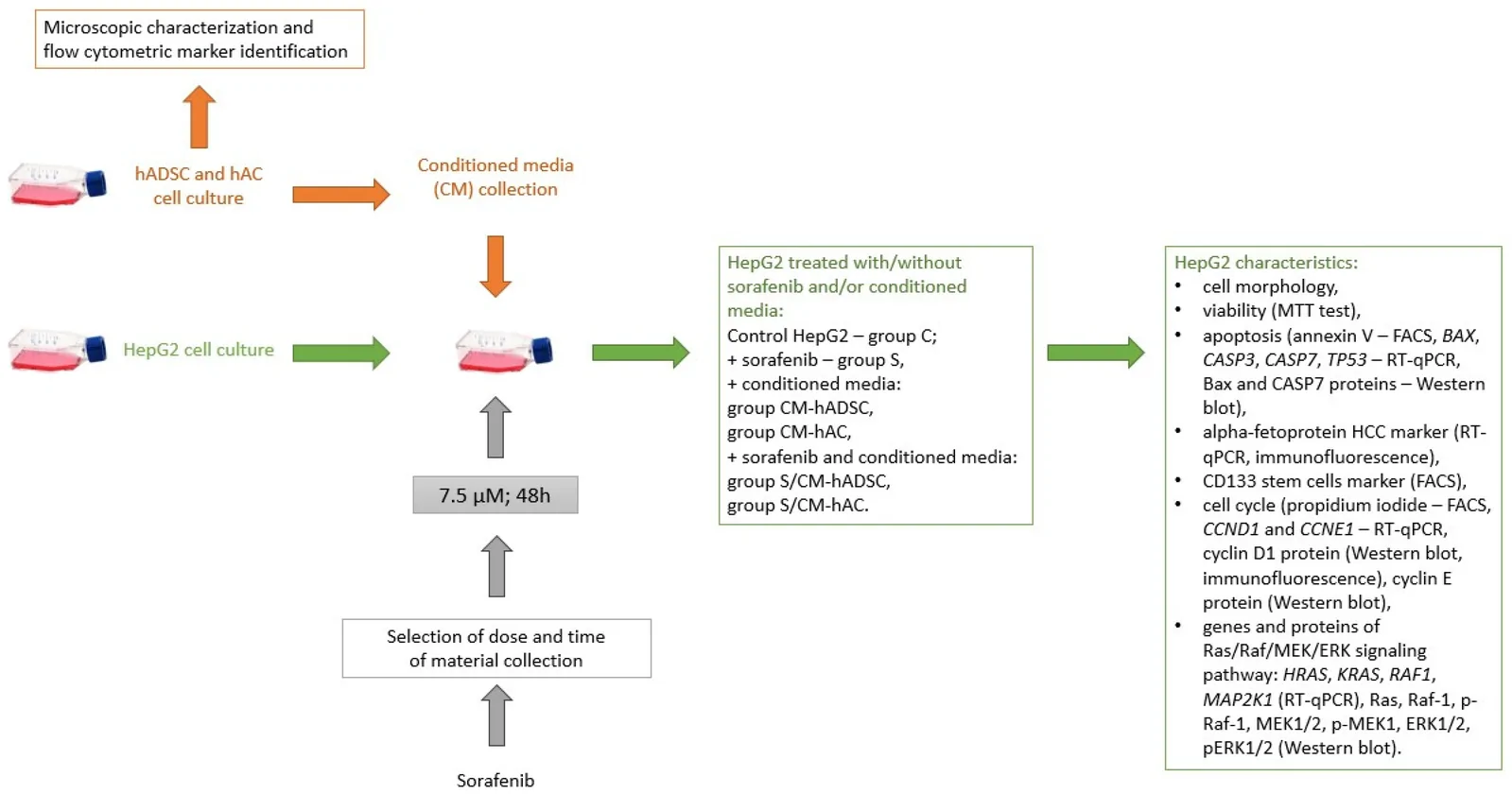
Experimental design. Based on preliminary studies, a sorafenib concentration of 7.5 μm and an exposure time of 48 h were selected for the main experiment.

In this study, HepG2 cancer cells were treated with hADSC- or hAC-derived CM and/or sorafenib (Figure 1). The sorafenib dose and time of material collection were chosen based on HepG2 cell viability at increasing sorafenib concentrations and exposition time. The study included the following groups: untreated HepG2 cells (group C, control) and HepG2 cells treated with sorafenib alone (group S), hADSC-derived CM (group CM-hADSC), CM-hADSC combined with sorafenib (group S/CM-hADSC), hAC-derived CM (group CM-hAC), and CM-hAC combined with sorafenib (group S/CM-hAC).

The study included the following stages:

1. hADSCs and hACs were cultured in standard medium (DMEM supplemented with 10% fetal bovine serum (FBS) and 1% penicillin–streptomycin–amphotericin B solution). Both cell types maintained their phenotype, in terms of morphology and surface markers, through three and seven subsequent passages, respectively.
2. CM from hADSCs and hACs were collected at 70% confluence.
3. Depending on the group, HepG2 cells were cultured under the following conditions:
  (a) with sorafenib at the selected dose of 7.5 μm and exposure time of 48 h;
  (b) in CM diluted 1:1 with standard medium for 48 h;
  or
  (c) in CM diluted 1:1 with standard medium supplemented with 7.5 μm sorafenib for 48 h.
4. After incubation, HepG2 cells were harvested and analyzed for viability (MTT assay), apoptosis (RT-qPCR and flow cytometry), cell cycle and cancer stem cell marker CD133 expression (flow cytometry), as well as AFP (HCC marker, RT-qPCR and immunofluorescence) and Ras/Raf/MEK/ERK pathway gene (RT-qPCR) and protein expression (Western blot and immunofluorescence).

### 2.2. Effective Dose and Exposure Time of Sorafenib

Sorafenib p-toluenesulfonate (LC Laboratories, Woburn, MA, USA) was dissolved in DMSO. HepG2 cells were treated with sorafenib at concentrations of 2.5, 5, 7.5, and 10 μm in culture medium for 24, 48, and 72 h. To determine the optimal dose and exposure time of sorafenib, HepG2 cell viability was assayed (MTT assay).

### 2.3. Stem Cell Cultures

The hADSC cell line was obtained from Lonza (Basel, Switzerland, cat. no. PCS-500-011). Amniotic cells were obtained from Applied Biological Materials Inc. (ABM; Richmond, BC, Canada, cat. no. T0531). Cells were cultured in standard medium consisting of Dulbecco’s Modified Eagle Medium (DMEM; Thermo Fisher Scientific, Waltham, MA, USA) supplemented with 10% FBS (EURx, Gdańsk, Poland), and 1% penicillin–streptomycin–amphotericin B solution (Corning, Glendale, AZ, USA). Cultures were maintained in a multi-gas incubator (Sanyo, Osaka, Japan) at 37 °C under 5% CO_2_. Cells were passaged at 70% confluence. Surface mesenchymal markers of hADSCs were evaluated at passage 3. The phenotype of hACs, defined by the presence of epithelial and MSC markers, was confirmed at passages 2 and 7.

#### 2.3.1. Characteristics of hADSCs and hACs

The morphology of hADSCs and hACs was evaluated at each passage using inverted microscopy. Additionally, cells were characterized by flow cytometry for the expression of MSC markers using mouse anti-human antibodies: PE-conjugated CD73, PE-Cy7-conjugated CD90, and APC-conjugated CD105 (Beckman Coulter, Brea, CA, USA). Mouse anti-human IgG1 (Beckman Coulter, Brea, CA, USA) were used as an isotype controls. hACs were further analyzed for the presence or absence of epithelial markers as cytokeratins 14, 15, 16, and 19 using a PE-conjugated antibody kit (BD Biosciences, Franklin Lakes, NJ, USA) (Table S1). Cells were suspended in staining medium (Beckman Coulter, Brea, CA, USA) and incubated with antibodies or isotype control for 30 min in the dark at room temperature. After incubation, cells were washed twice in staining medium and resuspended. The analysis was performed on a CytoFLEX flow cytometer (Beckman Coulter, Brea, CA, USA).

#### 2.3.2. Conditioned Media

To obtain conditioned media from hADSC and hAC cultures, cells were seeded into culture flasks at a density of 5 × 10^3^ cells/cm^2^ and maintained in complete growth medium at 37 °C in a humidified 5% CO_2_ incubator until they reached approximately 70% confluence. After 24 h, the medium was collected from hADSCs at passage 3 and from hACs at passage 7. The media were then centrifuged at 10,000× *g* for 10 min at 4 °C, filtered through a 0.2 µm filter to ensure removal of remaining particles, and diluted 1:1 with standard medium to ensure nutrient supply and optimal culture conditions for HepG2 cells during the 48 h incubation period.

### 2.4. HepG2 Phenotyping

The human HepG2 cell line was obtained from the American Type Culture Collection (ATCC, Manassas, VA, USA, cat. no. HB-8065). AFP, a marker of hepatocellular carcinoma, was assessed to confirm the HepG2 phenotype. The presence of cancer stem cell subpopulation was determined using the CD133 marker.

#### 2.4.1. Immunodetection of the CD133 Marker

The presence of the CD133 marker was confirmed in HepG2 cells using a PE-conjugated mouse anti-human antibody (R&D Systems, Minneapolis, MN, USA, cat no. FAB11331P) (Table S1). Cells were washed in PBS and incubated in 4% paraformaldehyde for 10 min. Then, cells were washed in PBS and incubated in 0.1% Triton X-100 solution in PBS for 10 min, resuspended in blocking buffer (1% bovine serum albumin (BSA), 10% normal goat serum, 0.3 M glycine in 0.1% PBS-Tween) for 30 min, followed by incubation with antibody or mouse IgG2a isotype control (R&D Systems, Minneapolis, MN, USA, cat no. IC003P) for 1 h in the dark at room temperature (Table S1). After incubation, cells were washed twice, resuspended in staining medium, and analyzed on a CytoFLEX flow cytometer (Beckman Coulter, Brea, CA, USA).

#### 2.4.2. Immunofluorescent Detection of Cyclin D and AFP Proteins

The expression of cyclin D and AFP was detected using immunofluorescence. HepG2 cells from the control and studied groups were seeded at a density of 50,000 cells per slide. Slides were fixed with 4% paraformaldehyde and incubated in blocking buffer (5% goat serum, Vector Laboratories, Newark, CA, USA) diluted in PBS (Corning, Glendale, AZ, USA) for 1 h at room temperature. BSA (Merck, Darmstadt, Germany) was diluted in PBS and used as the antibody dilution buffer. Then, cells were incubated overnight at 4 °C with primary antibodies: mouse anti-human Cyclin D (Proteintech, Rosemont, IL, USA, cat no. 60186-1-Ig) diluted 1:100, mouse anti-human AFP (Abcam, Cambridge, UK, cat no. ab3980) diluted 1:200, or mouse IgG1 isotype control (Abcam, Cambridge, UK, cat no. ab170190) diluted 1:200 for AFP and 1:66 for cyclin D (Table S1). After incubation, slides were washed twice in 0.2% Tween 20 (Sigma-Aldrich, Saint Louis, MO, USA) in PBS and incubated for 1 h with a secondary antibody (goat anti-mouse, dilution 1:1000, cat no. ab175473 for AFP and ab150133 for cyclin D, Abcam, Cambridge, UK) (Table S1). After washing twice in PBS, slides were stained with antifade mounting medium with DAPI (Vector Laboratories, Newark, CA, USA) and put on the glass slide. The immunohistochemical reactions were analyzed in a fluorescent microscope (BX43, Olympus, Tokyo, Japan) equipped with a camera (XC30, Olympus, Tokyo, Japan) running on the software cellSens Standard (Olympus, Tokyo, Japan).

#### 2.4.3. Evaluation of Gene Expression Associated with the Ras/Raf/MEK/ERK Pathway, Cell Cycle, and Apoptosis

RNA was isolated from HepG2 cells using the phenol/chloroform extraction with RNA Extracol (EURx, Gdansk, Poland), chloroform, isopropanol, and ethanol (all from Sigma-Aldrich, Saint Louis, MO, USA). The quality of RNA samples was evaluated using a NanoDrop 2000 instrument (Thermo Fisher Scientific, Waltham, MA, USA). The RT-qPCR reaction was performed using the GoTaq 1-Step RT-qPCR System (Promega, Madison, WI, USA) and a LightCycler 96 instrument (Roche, Basel, Switzerland). Primers for Ras/Raf/MEK/ERK pathway, cell cycle-related genes (Table S2) and apoptosis-related genes (Table S3) were obtained from Merck, Darmstadt, Germany. Beta-actin (*BACT*) was used as the reference gene.

#### 2.4.4. Semi-Quantitative Analysis of Selected Proteins

Expression of proteins related to the Ras/Raf/MEK/ERK pathway, cell cycle, and apoptosis was detected in HepG2 cells in the control and studied groups using Western blot. Cell pellet was lysed in RIPA Lysis Buffer (10×, Merck Millipore, Burlington, MA, USA) supplemented with cOmplete Mini Protease Inhibitor Cocktail (Roche, Basel, Switzerland) and a protease inhibitor cocktail (Sigma-Aldrich, Saint Louis, MO, USA). Lysates were then centrifuged (14,000 rpm, 15 min, 4 °C), and supernatants were collected. Next, solutions of 4× Laemmli Sample Buffer (Bio-Rad, Warsaw, Poland) and β-mercaptoethanol (Bio-Rad, Warsaw, Poland) were added, and the probes were incubated at 95 °C for 5 min.

Protein electrophoresis was performed using a PowerPac Basic Power Supply (Bio-Rad, Warsaw, Poland) in a 12% polyacrylamide gel with 15 µg of each protein sample. Protein transfer from the polyacrylamide gel to PVDF membrane was done using the wet electroblotting method. A 5% non-fat milk solution in TBST was used as blocking buffer for 1 h. The membranes were then incubated overnight at 4 °C with primary antibodies diluted in 5% non-fat milk solution. Analyses were conducted for proteins associated with the cell cycle, Ras/Raf/MEK/ERK signaling pathway, and apoptosis.

The following primary antibodies were used (Table S1): anti-cyclin E (mouse, Santa Cruz, Dallas, TX, USA) diluted 1:2500, anti-cyclin D1 (rabbit, Abcam, Cambridge, UK) diluted 1:10,000, anti-Ras (rabbit, Abcam, Cambridge, UK) diluted 1:5000, anti-Raf-1 (rabbit, Abcam, Cambridge, UK) diluted 1:1000, anti-p-Raf-1 (rabbit, Abcam, Cambridge, UK) diluted 1:1000, anti-MEK1/2 (rabbit, Abcam, Cambridge, UK), diluted 1:20,000, anti-p-MEK1 (rabbit, Abcam, Cambridge, UK) diluted 1:3000, anti-ERK1/2 (rabbit, Abcam, Cambridge, UK), diluted 1:10,000, anti-p-ERK1/2 (rabbit, Abcam, Cambridge, UK) diluted 1:10,000, anti-Bax (rabbit, Proteintech, Rosemont, IL, USA) diluted 1:8000, anti-cleaved caspase-3 (rabbit, Cell Signaling, Danvers, Massachusetts, USA) diluted 1:500, anti-caspase-7 (rabbit, Proteintech, Rosemont, IL, USA) diluted 1:600, anti-cleaved caspase-7 (rabbit, Abcam, Cambridge, UK) diluted 1:1000, anti-caspase-9 (rabbit, Proteintech, Rosemont, Illinois, USA) diluted 1:300. Reference proteins used in analysis were anti-GAPDH (rabbit, Sigma-Aldrich, Saint Louis, MO, USA) diluted 1:10,000 and anti-Hsp90 (rabbit, Abcam, Cambridge, UK) diluted 1:5000.

After incubation with primary antibodies, secondary antibodies (goat anti-rabbit, and goat anti-mouse, Abcam, Cambridge, UK) were diluted 1:10,000 in 5% non-fat milk solution and used to incubate proteins for 1 h at room temperature (Table S1). Proteins were visualized with SuperSignal West Pico PLUS chemiluminescent substrate (Thermo Fisher Scientific, Waltham, MA, USA) on an Invitrogen iBright CL750 Imaging System (Thermo Fisher Scientific, Waltham, MA, USA) using iBright image analysis software. Mean protein expressions were normalized to the expression of this protein in the control group and to the expression of GAPDH or Hsp90 proteins.

### 2.5. HepG2 Cell Viability

HepG2 cell viability was determined by the MTT assay and by flow cytometry using annexin V and propidium iodide (PI). The cell cycle of HepG2 cells was assessed by flow cytometry.

#### 2.5.1. MTT Assay

For MTT assay, HepG2 cells were seeded on a 96-well plate (5000 cells/well). Cells treated with 1% TritonX-100 served as a negative control, and cells cultured in standard medium served as a positive control. Following incubation, thiazolyl blue tetrazolium bromide (Sigma-Aldrich, Saint Louis, MO, USA, cat. no. M2128) was added. After three hours of incubation in 5% CO_2_ at 37 °C, formazan crystals were resuspended in DMSO, and samples were incubated for 15 min in the dark at room temperature. Samples were then analyzed at wavelengths of 540 nm and 690 nm using a Victor Nivo plate reader (PerkinElmer, Waltham, MA, USA).

#### 2.5.2. Cytometric Analysis of HepG2 Cell Viability and Apoptosis

The viability and apoptosis of HepG2 cells across all experimental groups were assessed using the PE Annexin V Apoptosis Detection Kit (BD, Franklin Lakes, NJ, USA). Cells were suspended in the binding buffer with annexin V and PI and incubated for 15 min in the dark at room temperature. After incubation, cells were resuspended in binding buffer. Flow cytometric analysis was performed using a CytoFLEX flow cytometer (Beckman Coulter, Brea, CA, USA). Cells were characterized as viable, early apoptotic, late apoptotic, and necrotic.

#### 2.5.3. Flow Cytometric Analysis of the Cell Cycle

The cell cycle of HepG2 cells was analyzed using FxCycle PI/RNase Staining Solution (Thermo Fisher Scientific, Waltham, MA, USA). Cells were fixed in 100% ethanol and washed in PBS. Then, cells were resuspended in FxCycle PI/RNase Staining Solution, incubated for 30 min in the dark at room temperature, and analyzed on a CytoFLEX flow cytometer (Beckman Coulter, Brea, CA, USA) to determine their assignment across the G1/G0, S and G2/M cell cycle phases. Results were analyzed using FCS Express software (De Novo Software, Pasadena, CA, USA).

### 2.6. Statistical Analysis

Statistical analysis was performed using TIBCO Statistica 13.3 software (TIBCO Software, Palo Alto, CA, USA). The Shapiro–Wilk test was used to assess the normality of distribution. For normally distributed variables, parametric tests were used (Student’s t-test and ANOVA with post hoc Tukey test). For variables with a non-normal distribution, non-parametric tests were used (Mann–Whitney test and Kruskal–Wallis test). A *p*-level of less than 0.05 was considered statistically significant.

## 3. Results

### 3.1. Effective Dose of Sorafenib and Culture Duration

The effective dose of sorafenib and culture duration were selected after assessing the effects of sorafenib concentrations ranging from 2.5 to 10 μm on the viability of HepG2 cells cultured in standard medium for up to 72 h.

**Figure 2:**
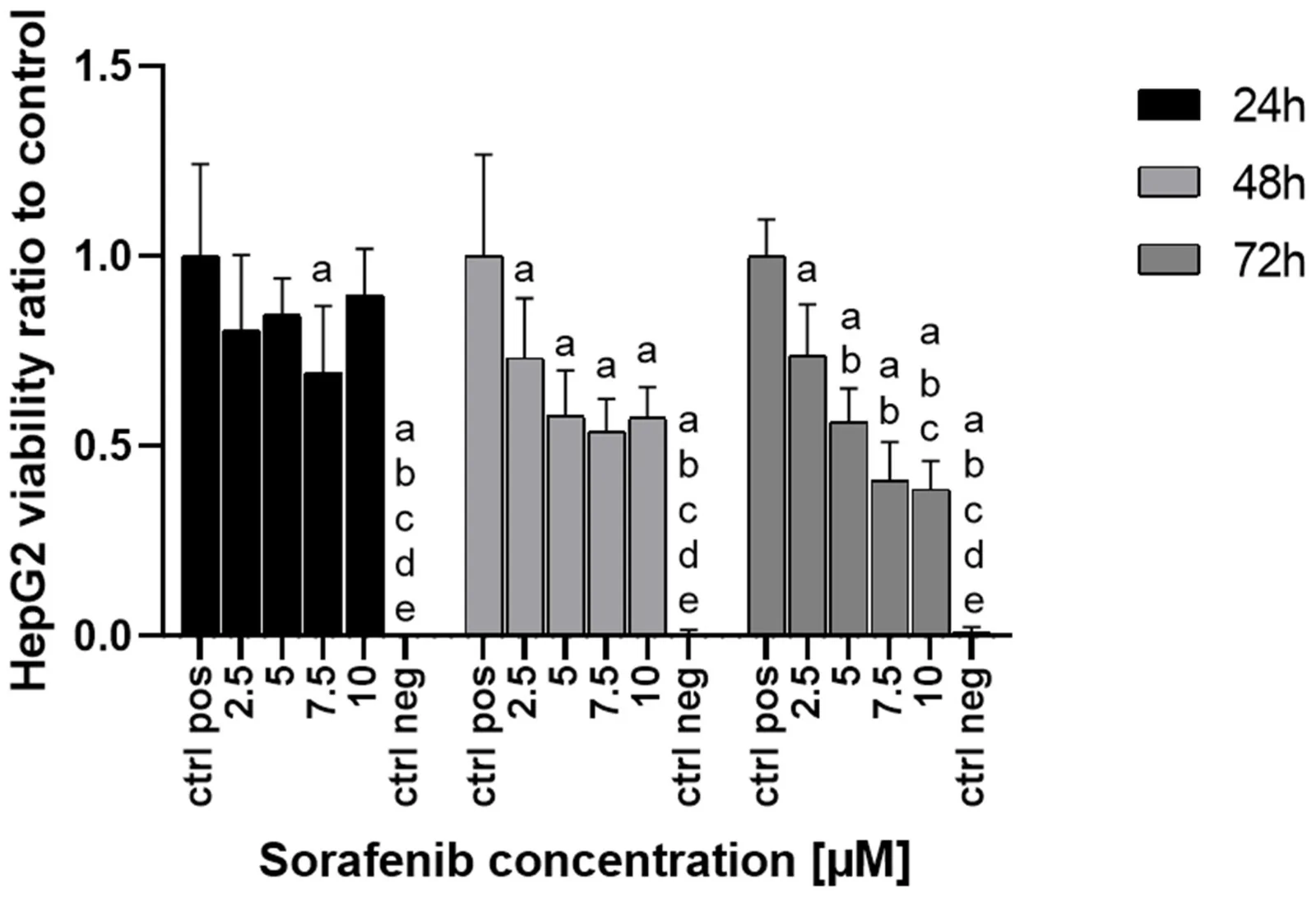
Effect of sorafenib concentrations on HepG2 cell viability (MTT assay). Each bar represents the ratio ± SD relative to the positive control (set as 1.0). Positive control (Ctrl pos)—cells cultured in standard medium. Negative control (Ctrl neg)—cells treated with 1% Triton X-100. Statistical significance (*p* < 0.05) as compared with: ^a^ positive control, ^b^ 2.5 μm, ^c^ 5 μm, ^d^ 7.5 μm, ^e^ 10 μm; *n* = 6.

For the 24 h incubation period, only the 7.5 μm dose significantly reduced HepG2 cell viability compared with the positive control (*p* < 0.05). After 48 h of incubation, cell viability was similarly reduced at all the tested concentrations (*p* < 0.05). However, after 72 h of incubation, cell viability decreased further in a dose-dependent manner, with comparable effects observed for the 7.5 and 10 μm concentrations (Figure 2).

Based on the results of cell viability analysis, a 7.5 μm dose of sorafenib with a 48 h exposure was selected for further experiments to determine its effects alone and in combination with CM on cultured HepG2 cells.

### 3.2. Phenotype of hADSCs and hACs

hADSCs and hACs displayed stable morphology up to passages 3 and 7, respectively. hADSCs were spindle-shaped, whereas the hAC population was morphologically heterogeneous, comprising both spindle-shaped and cuboidal cells (Figure S1). hADSCs were positive for MSC surface markers CD73 (99.11%), CD90 (99.43%), and CD105 (98.95%) (Figure S2). Similarly, hACs were positive for MSC surface markers in more than 98% of cells (Figure S3). In addition, cytokeratins were detected in 9.02–29.85% of hACs (Figure S3), indicating an intermediate phenotype with features of minor epithelial but predominantly mesenchymal lineages.

### 3.3. Comparison of the Effects of Sorafenib and Conditioned Media on HepG2 Cells

The effects of sorafenib (7.5 μm) and CM on the viability, apoptosis, cell cycle, and Ras/Raf/MEK/ERK pathway-related gene and protein expression in HepG2 cells were evaluated over a 48 h culture.

#### 3.3.1. Expression of CD133 and AFP

**Figure 3:**
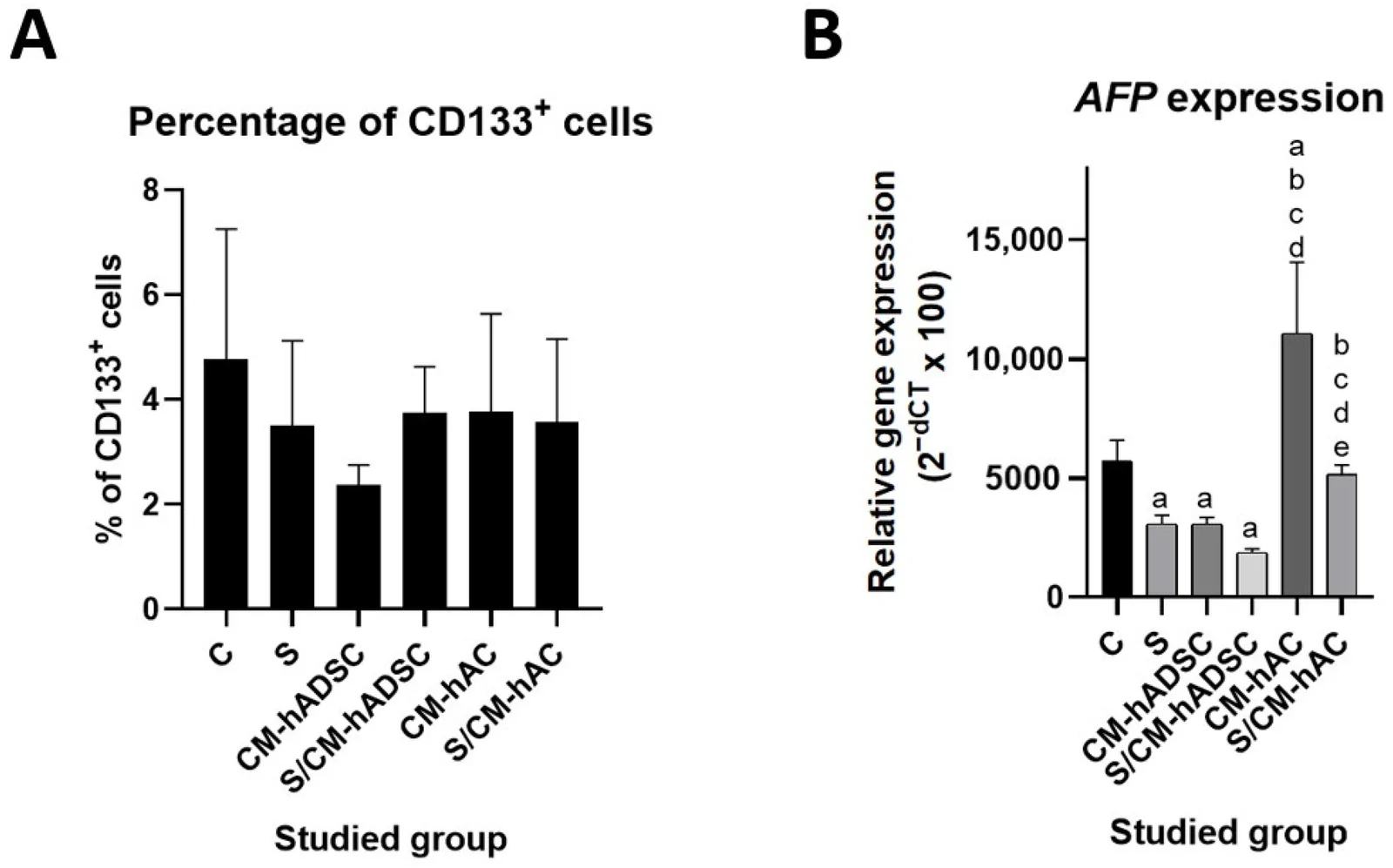
Assessment of CD133 and AFP markers. (**A**) Comparable percentage of CD133+ HepG2 cells in the control and sorafenib and/or CM-treated groups. Each bar represents the mean ± SD of CD133+ cells (*n* = 3). (**B**) Expression of *AFP* gene in HepG2 cells after incubation with 7.5 μm sorafenib and/or conditioned media. Each bar represents the ± SD (*n* = 9). Statistical significance (*p* < 0.05) as compared with: ^a^ C, ^b^ S, ^c^ CM-hADSC, ^d^ S/CM-hADSC, and ^e^ CM-hAC.

The mean percentage of CD133+ cells in the untreated C group was 4.77%. Although we observed a lower percentage of CD133+ HepG2 cells in the CM-hADSC group compared with the control, this tendency did not reach statistical significance (Figure 3A).

The expression of the *AFP* gene, a molecular marker of HCC, was decreased in the sorafenib-treated groups (S and S/CM-hADSC) and in the CM-hADSC group compared with the control (C) group (*p* < 0.05). In contrast, the CM-hAC group displayed significantly higher *AFP* expression compared with all other groups (*p* < 0.05). S/CM-hAC displayed this expression as relative to the C group and higher as compared to S, CM-hADSC and S/CM-hADSC groups. The results indicate that sorafenib alone and CM-hADSC, but not CM-hAC, inhibit *AFP* mRNA expression in HepG2 cells (Figure 3B).

To confirm the presence of AFP protein in all cultured HepG2 cells, immunofluorescence staining was performed (Figure S4).

#### 3.3.2. Viability of HepG2 Cells

**Figure 4:**
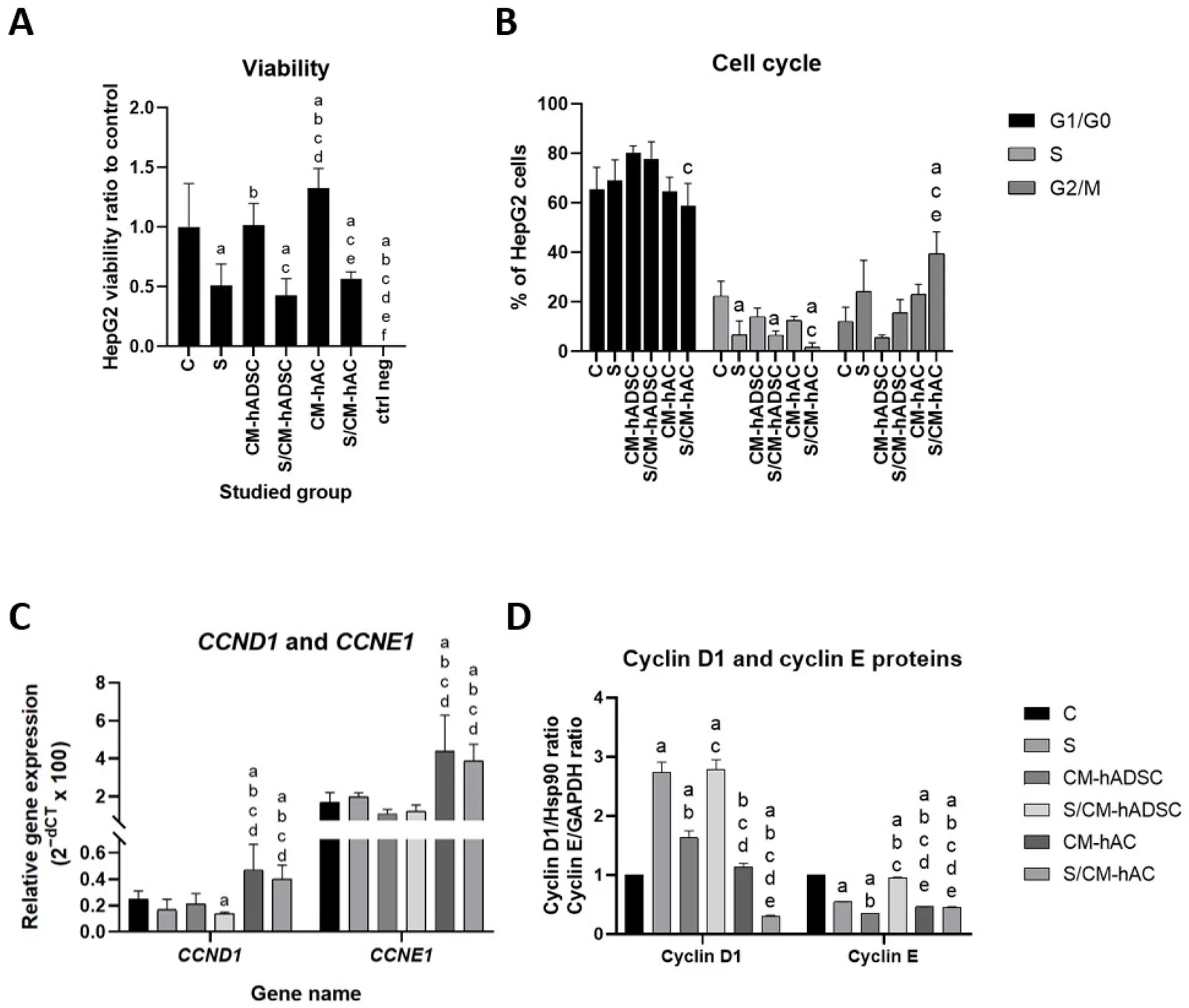
Viability and cell cycle progression of HepG2 cells after treatment with sorafenib and/or hADSC- or hAC-derived CM. (**A**) Viability of HepG2 cells (MTT assay). Ctrl neg—cells treated with 1% Triton X-100; *n* = 10. (**B**) Percentage of HepG2 cells in G1/G0, S, and G2/M cell cycle phases (flow cytometry); *n* = 3. (**C**) Expression of *CCND1* and *CCNE1* genes in HepG2 cells; *n* = 9. (**D**) Densitometric analysis of the expression of cyclin D1 and cyclin E proteins in HepG2 cells; *n* = 3. Each bar represents the mean ratio ± SD. Statistical significance (*p* < 0.05) as compared with: ^a^ positive control (C group), ^b^ S, ^c^ CM-hADSC, ^d^ S/CM-hADSC, ^e^ CM-hAC, and ^f^ S/CM-hAC.

Compared with the untreated group (C), 7.5 μm sorafenib reduced HepG2 cell viability in the S, S/CM-hADSC, and S/CM-hAC groups by 49.23%, 57.2% and 43.27%, respectively (*p* < 0.05). No significant difference was found between the S, S/CM-hADSC, and S/CM-hAC groups. HepG2 cell viability in the CM-hAC group was 1.33-fold higher (*p* < 0.05) than in the C group, 2.61-fold higher than in S group, 2.34-fold higher than in the S/CM-hAC group (*p* < 0.05), and 1.3-fold higher than in the CM-hADSC group (*p* < 0.05). The viability of HepG2 cells in the CM-hADSC group was comparable to the C group (*p* > 0.05) and was increased when compared with the groups containing sorafenib. These results suggest that CM-hAC supports HepG2 cell viability, whereas CM-hADSC has no significant effect. The inhibitory effect of sorafenib was not counterbalanced by any of the CM used (Figure 4A).

The cell cycle analysis included the percentages of cells in G1/G0, S, and G2/M phases (Figure 4B and Figure S5). The number of cells in the S phase was lower in the S, S/CM-hADSC, and S/CM-hAC groups compared with the C group (*p* < 0.05), suggesting an inhibitory effect of sorafenib that might influence DNA replication in some cells. The percentage of cells in the G2/M phase was higher in the S/CM-hAC group than in the C, CM-hADSC, and CM-hAC groups (*p* < 0.05).

*CCND1* and *CCNE1* mRNA expression in the CM-hAC and S/CM-hAC groups was significantly elevated (*p* < 0.05) (Figure 4C). Cyclin D1 protein expression was elevated in S, CM-hADSC, S/CM-hADSC and CM-hAC groups, but decreased in S/CM-hAC (*p* < 0.05). A decrease in cyclin E protein was observed in all groups (*p* < 0.05). The observed high *CCND1* mRNA and *CCNE1* mRNA expression after CM-hAC and S/CM-hAC administration was not fully reflected at the protein level (Figure 4D and Figure S6).

#### 3.3.3. Assessment of Pro-Apoptotic Effects of Sorafenib and Conditioned Media

**Figure 5:**
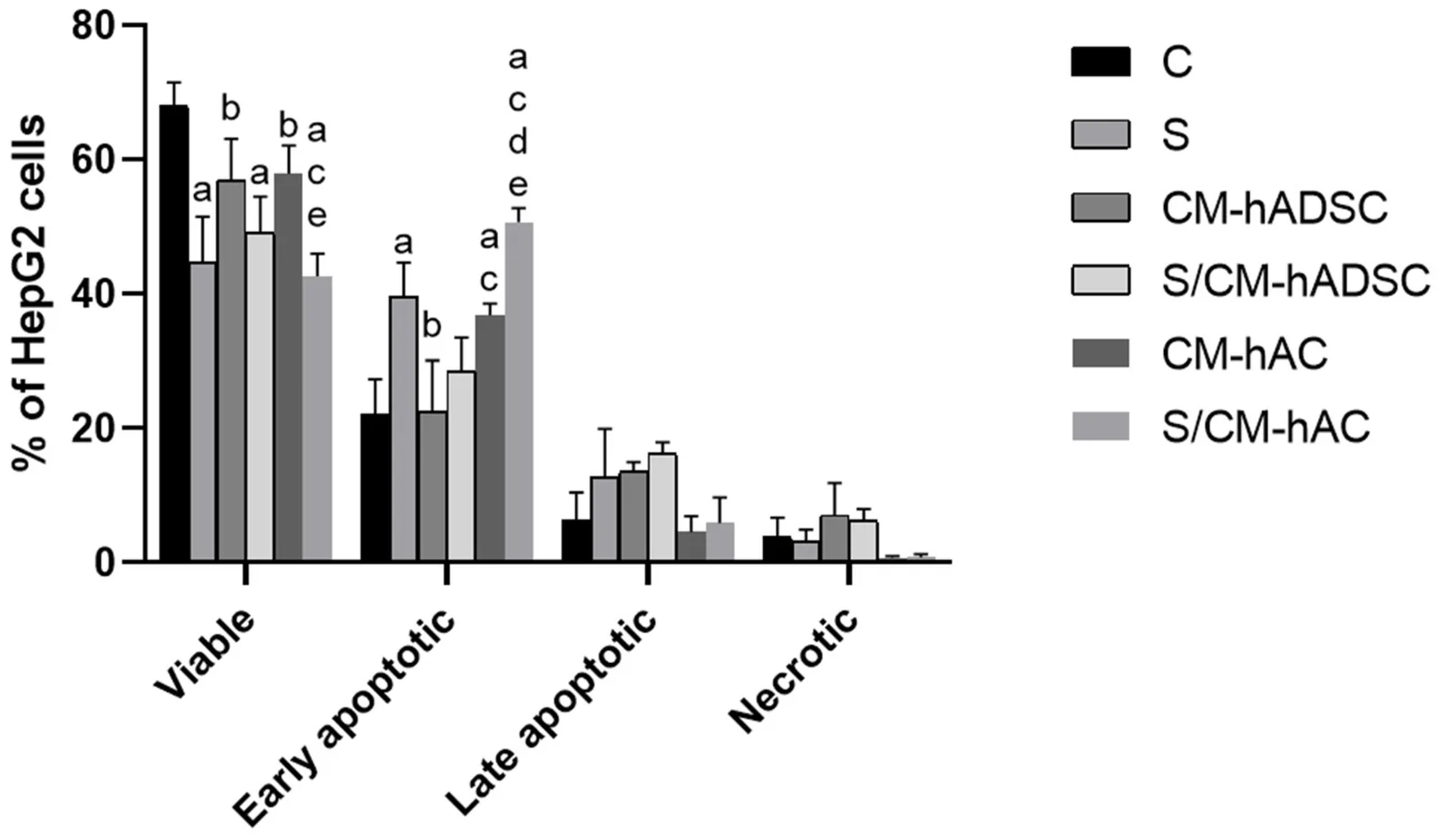
Percentages of viable, early apoptotic, late apoptotic, and necrotic HepG2 cells following incubation with sorafenib and hADSC-derived or hAC-derived conditioned media, administered alone or in combination. Each bar represents the mean ± SD. Statistical significance (*p* < 0.05) as compared with: ^a^ C, ^b^ S, ^c^ CM-hADSC, ^d^ S/CM-hADSC, and ^e^ CM-hAC; *n* = 3.

Significant changes were observed in the percentages of viable and early apoptotic HepG2 cells following incubation with sorafenib and/or CM, as demonstrated by cytometric measurements (Figure 5). Sorafenib reduced the percentage of viable cells in S, S/CM-hADSC, and S/CM-hAC groups compared with the C group (*p* < 0.05). Sorafenib increased the percentage of early apoptotic cells in S and S/CM-hAC groups (*p* < 0.05). CM alone did not affect the percentages of viable cells and had no effect (in the CM-hADSC) or increased the number of early apoptotic cells (in the CM-hAC group). In the S/CM-hAC group, the percentage of viable cells was lower, and the percentage of early apoptotic cells was higher, compared with the CM-hAC group (*p* < 0.05). The percentages of late apoptotic and necrotic cells did not change significantly in any group.

The above changes observed at the early stage of apoptosis corresponded with the observed changes in the expression of apoptosis-related genes, especially caspase genes. Although sorafenib itself did not statistically alter the expression of the *CASP3* gene or the *CASP7* gene, it should be considered that the expression of *CASP3* was noticeably increased. Interestingly, we observed that CM-hAC and S/CM-hAC significantly increased the expression of both *CASP3* and *CASP7* genes compared with the control groups and/or those treated with sorafenib and CM-hADSC (*p* < 0.05). On the other hand, the expression of the pro-apoptotic *BAX* gene, and *TP53*, which encodes the p53 protein that has the capacity to repair damaged DNA or induce apoptosis, did not differ statistically among the studied groups (Table 1).

**Figure 6:**
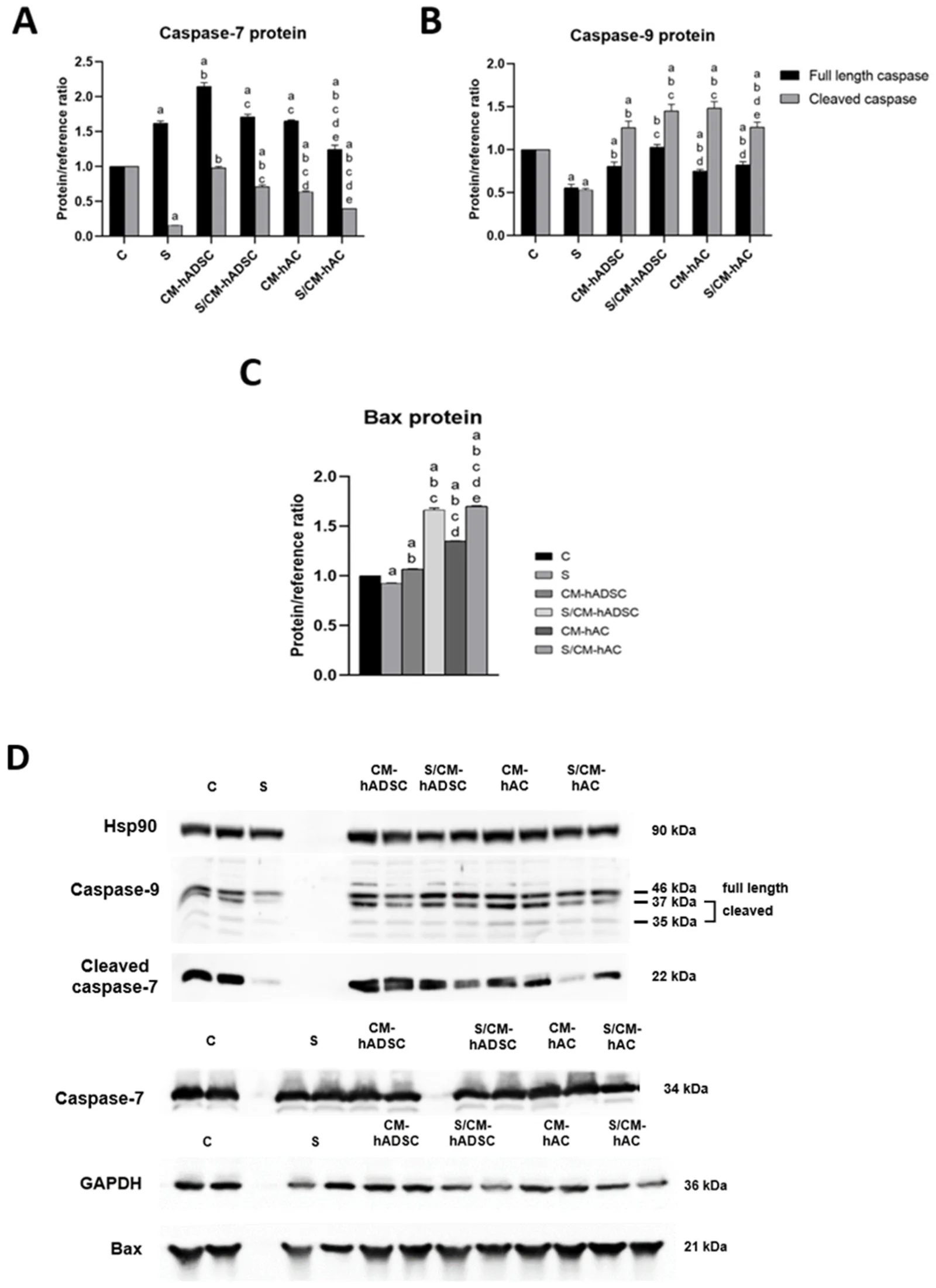
Apoptosis-related protein expression of HepG2 cells after treatment with sorafenib and/or hADSC- or hAC-derived CM. (**A**–**C**) Densitometric analysis of the expression of caspase-7, cleaved caspase-7, caspase-9, cleaved caspase-9 and Bax, respectively. Each bar represents the mean ± SD compared to control (C) = 1; *n* = 3. Statistical significance (*p* < 0.05) as compared with: ^a^ positive control (C group), ^b^ S, ^c^ CM-hADSC, ^d^ S/CM-hADSC, and ^e^ CM-hAC. (**D**) Representative visualization (Western blot).

Among the proapoptotic proteins (Figure 6), total effector caspase-7 increased in all experimental groups (*p* < 0.05), including group S (*p* < 0.05). Expression of cleaved forms of caspase-7 was decreased in all groups compared to the control group (*p* < 0.05), except for CM-hADSC, and relative to full-length forms in all groups. Total caspase-9 was found to be downregulated in all groups (*p* < 0.05) except S/CM-hADSC. Its cleaved active form exhibited increased expression in most groups (*p* < 0.05) except group S. Bax protein expression increased in most experimental groups (*p* < 0.05), especially in the groups treated with CM and S combined, but excluding the group treated with S alone.

#### 3.3.4. Expression of Ras/Raf/MEK/ERK Pathway-Related Genes and Proteins

The inhibitory effect of sorafenib on the Ras/Raf/MEK/ERK signaling pathway genes was mainly manifested by a reduction in the expression of *RAF1* and *MAP2K1* genes (*p* < 0.05) (Table 2). Statistically, this tendency was also maintained in the S/CM-hADSC. What is more, CM-hAC alone noticeably increased these two expressions as compared to the S group, which was a difference as compared to CM-hADSC alone.

**Figure 7:**
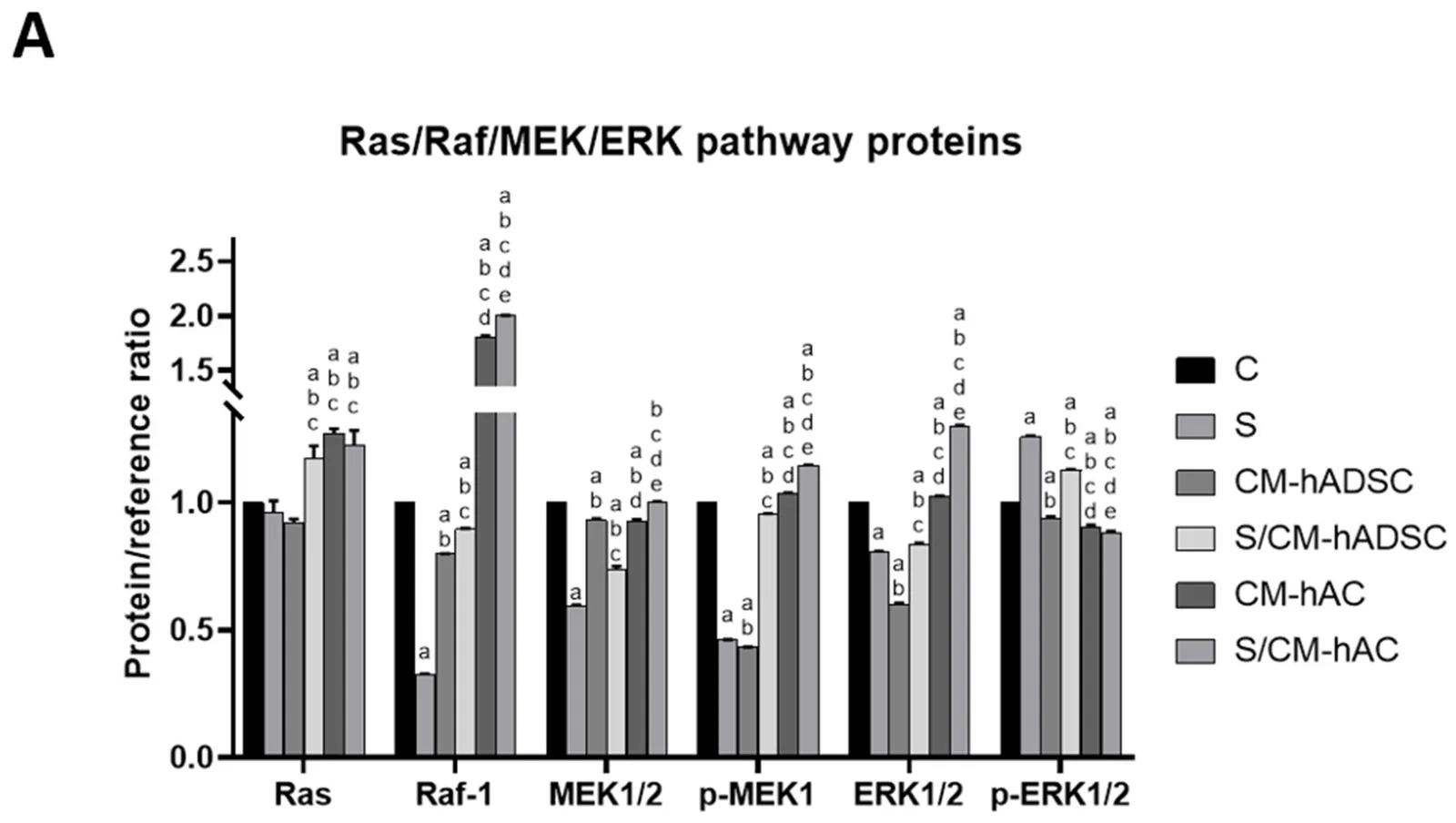

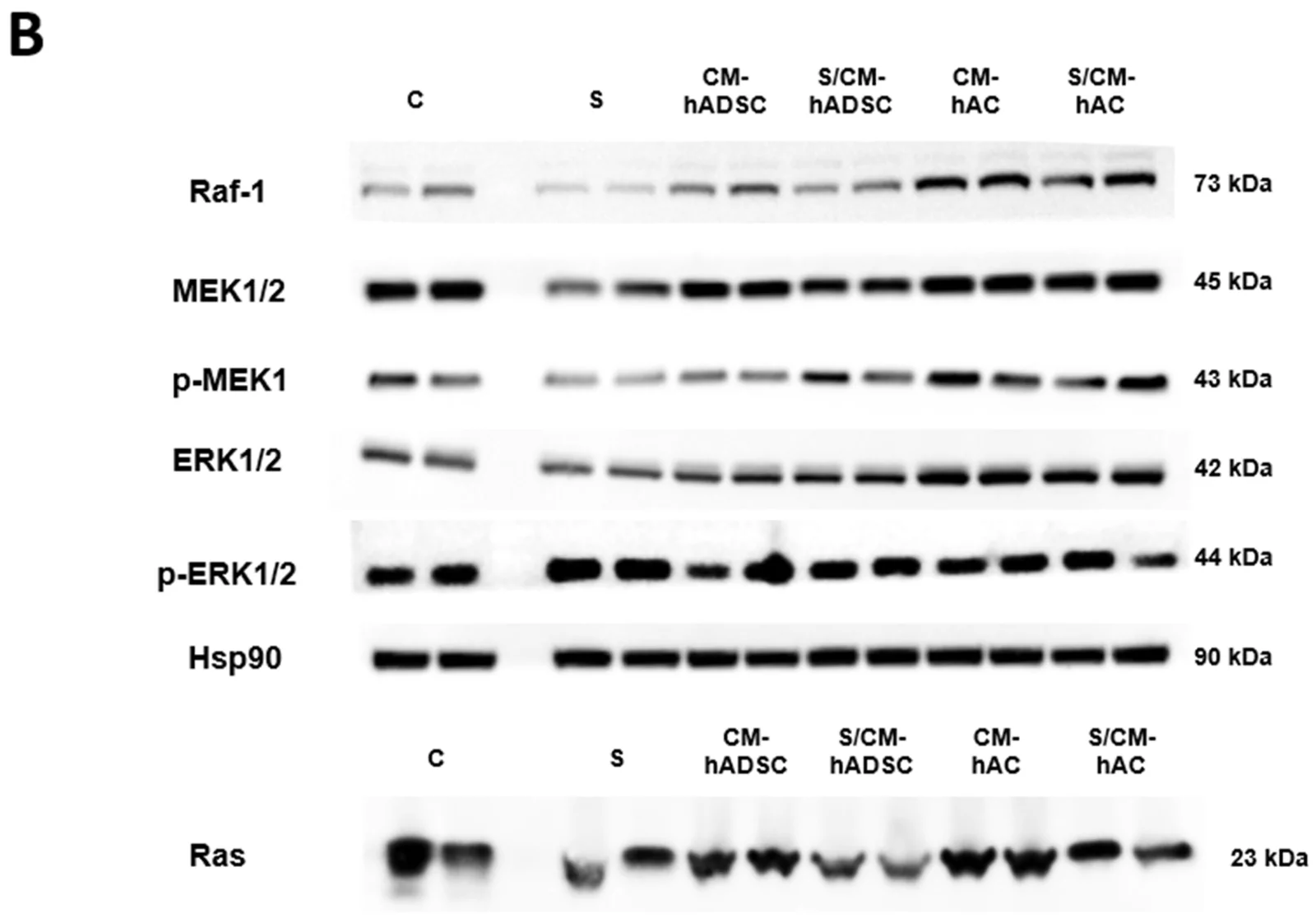
Expression of Ras/Raf/MEK/ERK pathway proteins in HepG2 cells after treatment with sorafenib and/or hADSC- or hAC-derived CM. (**A**) Densitometric analysis of Ras, Raf-1, p-Raf-1, MEK1/2, p-MEK1, ERK1/2 and p-ERK1/2 protein expression. Each bar represents the mean ± SD compared to control (C) = 1; *n* = 3. Statistical significance (*p* < 0.05) as compared with: ^a^ C, ^b^ S, ^c^ CM-hADSC, ^d^ S/CM-hADSC, and ^e^ CM-hAC; *n* = 3. (**B**) Representative immunoblots corresponding to total and phosphorylated Ras/Raf/MEK/ERK pathway proteins. p-RAF-1 expression was undetectable.

Under the influence of sorafenib Ras expression was unchanged, but a significant (*p* < 0.05) reduction in the expression of Raf-1, MEK1/2, p-MEK1, and ERK1/2 proteins (by 19.16% to 67.2%), and increased p-ERK1/2 expression by 25.64% (*p* < 0.05), were observed in HepG2 cells. Ras protein expression was also unchanged by CM-hADSC, but the expression of all other proteins of the Ras/Raf/MEK/ERK pathway, including phosphorylated forms, was significantly reduced by this CM compared to control, and at a lower (Raf-1, MEK1/2) or comparable (p-MEK1, ERK1/2) degree to sorafenib. Also, the combined administration of sorafenib and CM-hADSC resulted in a decrease in the expression of almost all proteins of the pathway compared to the control (except for Ras and p-ERK1/2, where the expression increased). A clear inducing effect of CM-hAC on the expression of Ras, Raf-1, p-MEK1, and ERK1/2, and of S/CM-hAC on the expression of Ras, Raf-1, p-MEK1, and ERK1/2, as well as their inhibitory effect on the expression of MEK1/2 and/or p-ERK1/2, was observed (Figure 7).

## 4. Discussion

The Ras/Raf/MEK/ERK pathway plays a crucial role in HCC cell survival, proliferation, and metabolism [3]. In HCC, genes related to this pathway are often mutated, and the overall pathway activity is upregulated [3,4]. Blocking the Ras/Raf/MEK/ERK signaling pathway is the main mechanism underlying the anticancer activity of sorafenib in HCC. Clinically, patients eligible for sorafenib therapy receive 800 mg of the drug per day, administered orally [8]. In in vitro studies, the typical sorafenib concentration ranges from 5 to 10 µM [11,50,51], and we selected 7.5 μm for our experiments. It has also been suggested that hADSCs produce certain factors, such as miR-100-5p, which may affect the activity of the Ras/Raf/MEK/ERK signaling pathway [52]. We hypothesized that adipose tissue and amnion-derived stem cells expressing mesenchymal markers produce a secretome that may enhance the effect of sorafenib, a standard drug used in HCC, by promoting apoptosis and inhibiting the Ras/Raf/MEK/ERK signaling pathway.

High viability and the ability to self-renew are fundamental features of cancer cells, enabling malignant tumors to grow and survive. Sorafenib, a classic drug used in advanced HCC, reduces cell viability not only in the HepG2 cell line [53,54,55], but also in Hep3B, Huh7, and PLC/PRF/5 HCC cell lines [11,56]. Furthermore, it has been suggested that sorafenib causes cell cycle arrest by blocking the G2/M phase [53,54] following DNA replication, thereby inhibiting cell division. In line with these observations, we observed a significant reduction in the number of viable cells under the influence of sorafenib, which was accompanied by a significant decrease in the proportion of cells in the S phase of the cell cycle, which plays a key role in DNA replication. As anticipated, the anti-proliferative effect of sorafenib was confirmed. It was also manifested in higher cyclin D1 and lower cyclin E protein expression. The latter effect likely corresponded to protein degradation via post-translational modification via the ubiquitin-proteasome system after peak cyclin E expression in G1/S phase, followed by degradation in S phase [57]. In this process, phosphorylated cyclin E is ubiquitinated and targeted to the proteasome for degradation [58,59]. Therefore, even when *CCNE1* mRNA is high (as in the group CM-hAC and S/CM-hAC) or unchanged (in other groups), a decrease in protein levels can be observed. Post-translational regulation of cyclin E has been observed in human and non-human cells [60]. It has been demonstrated that cyclin E is often overexpressed in many malignant cells, which correlates with excessive cell survival [61,62]. Low level of cyclin E in cancer cells cultured with hAM-MSCs has been observed [63].

Similarly to cyclin E gene/protein expression, we observed a lack of cyclin D1 mRNA-protein synchronization with a predominantly increased expression of cyclin D1 protein in most experimental groups, especially in the S and S/CM-hADSC groups. This lack of synchronization has been previously described in multiple myeloma cells [64]. It has also been suggested that the higher cyclin D1 protein expression in HCC cells after sorafenib treatment may result from activation of the PI3K/AKT cascade as an escape mechanism [65]. Paradoxical upregulation of cyclin D1 expression by sorafenib has also been described in non-small cell lung cancer cells [66].

Reduced HepG2 cell viability during sorafenib treatment was also manifested by decreased expression of AFP, a clinical marker overexpressed in HCC [1,67], with unchanged numbers of cells expressing CD133 (a marker of cancer stem cells, a subpopulation that contributes to sorafenib resistance [68]). CM from hAC cells, which have a partially epithelial phenotype, increased *AFP* mRNA expression, whereas CM-hADSC derived from mesenchymal cells did not. The concomitant increased cell viability in the CM-hAC group was examined using the MTT assay, which reflects mitochondrial metabolic activity rather than directly cell number. At the same time, the percentage of viable HepG2 cells in the CM-hAC group (about 58%), although lower, was not statistically different from the control group, while the number of cells in early apoptosis increased to less than 37% and was accompanied by *CASP3* and *CASP7* mRNA overexpression. This effect was enhanced by the combined administration of CM-hAC and sorafenib. Therefore, although the activity of both CM did not offset the inhibitory effect of sorafenib on the number of viable tumor cells, the effect of CM-hAC was likely related to the early induction of apoptosis in a subset of cells in culture and the concomitant increase in *AFP* expression. We did not observe significant changes in the number of cells in advanced apoptosis in either experimental group. It was observed that high *AFP* expression is associated with tumor progression or dedifferentiation [69,70]. High *AFP* mRNA expression can reverse the proapoptotic effect of chemotherapy [71] and inhibit apoptosis [72]. Silencing *AFP* mRNA expression induced apoptosis and increased sensitivity to sorafenib in HepG2 cells [72]. In this context, the potential use of the hAC secretome in the clinic could pose a significant risk of promoting tumor growth and progression, thus excluding it as a potential anticancer agent. In this light, it must be considered that the relationship between increased *AFP* expression and cell apoptotic activity may be more complex, given that we simultaneously observed increased viability of HepG2 cells.

Induction of apoptosis is one of the key mechanisms of action of drugs used in cancer treatment. The evasion of the natural mechanism of apoptosis by cancer cells allows them to pass on mutations to subsequent generations. The apoptotic process can be initiated by caspase-dependent or -independent signaling pathways. In both cases, the pro-apoptotic protein Bax plays a functional role [73]. Caspase-9 has been identified as an initiator of the intrinsic apoptotic pathway, while caspases-3 and -7 act as effectors, with their cleaved forms being considered active [73,74].

Sorafenib has been shown to induce apoptosis in HCC cell lines [11,75]. Most authors assume that sorafenib activates the caspase-dependent apoptosis pathway in HepG2 cells, resulting in an increase in the cleaved, active forms of these proteases as well as Bax protein [76,77,78]. However, we did not demonstrate an inducing effect of sorafenib on either the mRNA expression of caspase-3 and -7 or the level of cleaved forms of caspase-7 and caspase-9, and Bax protein. It cannot be excluded that a caspase-independent signaling pathway may play a significant role in this experimental setup. In addition to caspase cleavage, other factors influence the apoptotic process, including metabolic activity and cell membrane permeability [74,79]. Some researchers have noted an alternate pattern in the data regarding caspase protein expression. Sorafenib demonstrated no alteration in the expression of cleaved caspase-3 and -8 [80], and in one study even a decrease in cleaved caspase-3 was observed [81]. It downregulated caspase-1 expression in a dose-dependent manner [82]. One study revealed that although sorafenib elevated the expression levels of cleaved caspase-3, -8 and -9, the increase in cleaved caspase-9 expression was significant only at higher doses [77].

In this context, it is important to note that during apoptosis, changes in the cell membrane composition occur, including the transition of phosphadytyloserine from the inner to the outer surface of the cell membrane [83]. This phenomenon is exploited in Annexin V staining to detect apoptosis, where the dye binds to phosphadytyloserine on the outer surface of the membrane [84]. Cells stained with annexin V and subsequently detected by flow cytometry are then defined as a population of early apoptotic cells. Co-application of propidium iodide allows for the distinction between viable, apoptotic, and necrotic cells [84]. It has been suggested that the exposure of phosphadytyloserine on the outer surface of the cell membrane is associated with caspase cleavage [83]. However, it has been shown that phosphadytyloserine can be exposed in cells even after the use of caspase inhibitors [85] or after *CASP9* knockdown [86], thus confirming a potential caspase-independent process of cell death with features resembling apoptosis. One study demonstrated caspase-independent phosphadytyloserine exposure, measured as annexin V binding, following sorafenib treatment of breast cancer cells [87]. In our flow cytometry study, we observed early sorafenib-induced apoptosis identified by annexin V staining without concomitant caspase activation. We therefore suggest that in this experimental setting, sorafenib induced caspase-independent apoptosis in HepG2 cells or that apoptosis was observed early in the process and, therefore, the caspase cascade was not yet observed.

On the other hand, in the present study, we observed decreased expression of cleaved caspase-7 and increased expression of cleaved caspase-9 with a significant increase in Bax protein in all experimental groups of HepG2 containing CM. The observed increase in caspase expression at the mRNA level under the influence of hAC-CM was not confirmed by the expression of the corresponding proteins and their cleaved forms. Nevertheless, considering the observed changes in the number of early apoptotic cells, it can be assumed that hAC-derived CM could enhance apoptosis induction, although the caspase-dependent mechanism of this process remains to be elucidated. The results of studies in which the pro-apoptotic effect of CM-hADSC or CM-hAC has been observed may serve as a point of reference [43,88]. Specifically, co-culture with hADSCs or CM-hADSC induced a pro-apoptotic effect in HepG2 cells [32,33]. A similar effect was observed for hAM-MSCs and hAM-MSC-derived CM [43].

Finally, the Ras/Raf/MEK/ERK pathway is crucial in the development of HCC, and most patients have mutations or overexpression of its genes [4,5,6]. This pathway, among others, participates in the regulation of cell proliferation and apoptosis [3,6]. Blocking this pathway is a major mechanism of sorafenib action in both HCC and renal cell carcinoma [14]. Previously used doses of sorafenib of 5 and 10 µM effectively inhibited the Ras/Raf/MEK/ERK pathway in HepG2 cells [51]. We observed that sorafenib at a dose of 7.5 µM inhibited the *RAF1* and *MAP2K1* genes, which belong to the Ras/Raf/MEK/ERK pathway. CM-hAC, administered alone or in combination with sorafenib, stabilized *RAF1* and *MAP2K1* gene expression at control levels to a significantly greater extent than CM-hADSC. By measuring the levels of both phosphorylated and non-phosphorylated proteins within the pathway, sorafenib was observed to inhibit this pathway. The inhibitory effect was counterbalanced by CM, particularly CM-hAC. CM-hAC was hypothesized to activate the pathway, as evidenced by increased levels of Ras, Raf-1, ERK1/2, and p-MEK1. However, sorafenib increased p-ERK1/2, the pathway’s effector protein. The paradoxical increase in p-ERK after sorafenib treatment has been previously described in HCC [89] and other cancer cells [90], and may be linked to sorafenib resistance [89]. The suppression of p-ERK has been shown to be effective in overcoming sorafenib resistance in HCC [91]. p-ERK expression may change over time in cancer cells exposed to sorafenib [92], increasing in response to prolonged exposure after 48 h [93].

Although the crucial role of the Ras/Raf/MEK/ERK pathway in HCC pathogenesis, as well as in cell survival and proliferation, has been confirmed [94], it should be taken into consideration that other pathways are also important in apoptosis induction and proliferation inhibition [95], such as PI3K/AKT [96], which plays a crucial role in inflammatory response [97]. Moreover, there are some doubts about the suitability of HepG2 cells as a model of HCC, because some studies may suggest that the HepG2 cell line represents hepatoblastoma rather than hepatocellular carcinoma [98,99]. Hepatoblastoma represents a distinct clinic entity with a different molecular pattern, with Wnt/beta-catenin pathway regarded as being of pivotal significance [100]. Although some studies have reported the presence of alterations in components of the Ras/Raf/MEK/ERK pathway in hepatoblastoma [101] and suggested a role in tumor progression [102], this signaling axis is generally not considered the primary oncogenic driver in this disease. Therefore the potential data on CM influence on Ras/Raf/MEK/ERK pathway might be analyzed; however, it could be less relevant in clinical context. In such a case, changes in the gene expression of the Ras/Raf/MEK/ERK pathway may not fully correspond to changes in the viability or apoptotic activity of HepG2 cells.

To summarize, sorafenib significantly reduced HepG2 cell viability, the proportion of viable to apoptotic cells, and the percentage of cells in the S phase of the cell cycle. It also contributed to reduced *AFP* gene expression. The effect of sorafenib on reducing cell viability and increasing early apoptosis was associated with decreased expression of most genes of the Ras/Raf/MEK/ERK pathway, excluding p-ERK1/2, with decreased Bax protein expression and cleaved forms of caspase-7 and caspase-9. The role of caspases in the mechanism of apoptosis induction in HepG2 cells requires further clarification. Due to uncertainties regarding the nature of HepG2 cells in the context of and their relationship with hepatoblastoma and HCC, comparative studies should be conducted on several cancer cell lines.

Conditioned media obtained from hADSCs and hACs showed partially distinct effects on HepG2 cells as compared to sorafenib. Unlike sorafenib, CM did not reduce HepG2 viability significantly. The simultaneous effect of CM-hAC on gene stabilization in the Ras/Raf/MEK/ERK pathway was more pronounced than that of CM-hADSC, regardless of the presence of sorafenib, and was accompanied by an increase in caspase gene expression and a greater number of early apoptotic cells. Approximately 37% of HepG2 cells entered the early phase of apoptosis upon exposure to CM-hAC (which is comparable to that following sorafenib alone), and even more following combined administration of sorafenib and CM-hAC (>50%). It can also be assumed that CM may influence the activity of sorafenib by modulating proteins involved in drug transport, metabolism and accumulation [103].

Functional differences between the hACs used in this study and hADSCs resulting from different phenotypes may explain the differences in the effect of appropriate CM on gene and protein expression levels. Differences in the proteomic profile of hADSCs and amnion-derived epithelial and mesenchymal cells exhibiting divergent phenotypes may be an important reason for the different effects of their secretomes on other cells [47,104,105].

## 5. Conclusions

1. The effect of sorafenib on HepG2 cells, reducing its viability, inhibiting the cell cycle, decreasing the expression of the *AFP* gene and most proteins related to the Ras/Raf/MEK/ERK pathway, was most often dominant over CM when administered together with one of them.
2. The effect of sorafenib on increasing the number of early apoptotic HepG2 cells was not associated with increased Bax protein expression and the participation of cleaved forms of caspase-7 and caspase-9.
3. The effect of CM-hAC was often different from that of CM-hADSC and consisted of enhancing the inhibitory effect of sorafenib at the cell cycle level and increasing the number of early apoptotic cells and caspase mRNA expression, while activating the Ras/Raf/MEK/ERK signaling pathway and inducing *AFP* gene expression.
4. The effects of sorafenib and CM derived from different types of stem cells may differ significantly, meaning that the role of the stem cell secretome as a potential anticancer therapy requires further investigation, taking into account different types of cancer cell lines and detailed analysis of the secretome composition.

## Figures and Tables

**Table 1:** Expression of apoptosis-related genes (*BAX*, *TP53*, *CASP3*, and *CASP7*) in HepG2 cells treated with 7.5 μm sorafenib, administered alone or in combination with CM-hADSC or CM-hAC. The values represent the medians of 2^−dCT^ × 100, with first and third quartiles. Statistical significance (*p* < 0.05) as compared with: ^a^ C, ^b^ S, ^c^ CM-hADSC, and ^d^ S/CM-hADSC; *n* = 9.

	*BAX*	*TP53*	*CASP3*	*CASP7*
**C**	21.4 (7.25–24.73)	4.67 (1.44–7.15)	9.31 (8.8–10.25)	1.22 (0.82–1.39)
**S**	15.08 (7.4–22.76)	4.71 (2.62–7.35)	16.77 (14.3–19.4)	0.8 (0.76–1.05)
**CM-hADSC**	14.26 (12.59–16.27)	5.59 (5.40–5.87)	5.79 (4.8–6.52) ^b^	0.8 (0.74–1.14)
**S/CM-hADSC**	18.77 (13.27–20.54)	5.35 (5.17–6.97)	10.47 (7.79–12.05)	1.11 (0.96–1.25)
**CM-hAC**	20.06 (11.85–21.21)	2.67 (2.59–4.16)	28.57 (23.86–37.96) ^a,c,d^	1.74 (1.64–2.8) ^a,b,c^
**S/CM-hAC**	12.95 (12.17–19.9)	4.84 (4.42–5.75)	37.91 (28.14–40.36) ^a,c,d^	1.53 (1.21–1.61) ^b^

**Table 2:** Expression of Ras/Raf/MEK/ERK pathway-related genes: *HRAS*, *KRAS*, *RAF1*, and *MAP2K1* in HepG2 cells after incubation with 7.5 μm sorafenib administered alone or in combination with CM derived from hADSCs or hACs. The values represent the medians of 2^−dCT^ × 100, with first and third quartiles. Statistical significance (*p* < 0.05) as compared with: ^a^ C, ^b^ S, ^c^ CM-hADSC, and ^d^ S/CM-hADSC; *n* = 9.

	*HRAS*	*KRAS*	*RAF1*	*MAP2K1*
**C**	0.3 (0.12–0.4)	0.93 (0.36–1.43)	0.26 (0.2–0.33)	30.66 (17.98–34.49)
**S**	0.2 (0.14–0.39)	0.77 (0.47–0.94)	0.17 (0.15–0.2) ^a^	13.2 (11.38–23.92) ^a^
**CM-hADSC**	0.25 (0.21–0.31)	0.94 (0.8–1.03)	0.14 (0.12–0.2)	22.07 (20.03–23.82) ^b^
**S/CM-hADSC**	0.25 (0.21–0.37)	1.06 (0.8–1.13)	0.15 (0.13–0.21)	14.33 (12.99–14.78) ^a^
**CM-hAC**	0.22 (0.19–0.49)	0.74 (0.59–1.27)	0.42 (0.32–0.53) ^b,c,d^	30.41 (21.8–67.01) ^b,d^
**S/CM-hAC**	0.28 (0.27–0.33)	1.06 (1–1.24)	0.26 (0.25–0.35) ^b^	29.34 (26.81–34.41) ^b,d^

## Data Availability

Data is provided within the manuscript.

## Supplementary Materials

The following supporting information can be downloaded at: https://www.mdpi.com/article/10.3390/jcm15176738/s1, Table S1: Primary and secondary antibodies and isotype controls used for flow cytometry (FACS), immunohistochemistry (IHC), and Western blot (WB); Table S2: Primers for Ras/Raf/MEK/ERK pathway and cell cycle-related genes used in RT-qPCR; Table S3: Primers for *AFP* and apoptosis-related genes used in RT-qPCR; Figure S1: Cell cultures in standard medium. (A) Spindle-shaped human adipose tissue-derived mesenchymal stem cells (hADSCs). (B) Human amnion-derived cells (hACs) consisting of spindle-shaped and cuboidal cells. Scale bar = 200 µm; Figure S2: Expression of mesenchymal surface markers on hADSCs (passage 3). The percentage of cells expressing individual markers is given. UC—unstained control; Figure S3: Expression of epithelial and mesenchymal surface markers in hACs. A—passage 2, B—passage 7. The percentage of cells expressing individual markers is given. CK—cytokeratins; IC—isotype control; UC—unstained control; Figure S4: Immunodetection of cyclin D (left) and AFP (right) proteins in HepG2 cells treated with sorafenib and/or CM-hADSC and CM-hAC. Nuclei were stained with DAPI. Scale bar = 20 μm; Figure S5: Comparison of cell cycle phase distribution in HepG2 cells after treatment with sorafenib and/or CM. G1/G0—first peak; G2/M—second peak; S—between the first and second peaks; Figure S6: Representative visualization (Western blot) of cyclin E and cyclin D1 proteins in HepG2 cells after culture with sorafenib and/or hADSC- or hAC-derived CM.

## Abbreviations

The following abbreviations are used in this manuscript:

AFP	Alpha-fetoprotein
CM	Conditioned medium
hACs	Human amnion-derived cells expressing epithelial and mesenchymal markers
hADSCs	Human adipose tissue-derived stem cells
hAECs	Human amniotic epithelial cells
hAM-MSCs	Human amniotic mesenchymal stem cells
HCC	Hepatocellular carcinoma
