# Supplementary material for: Comparison of the Effects of Sorafenib and Stem Cell Secretome on HepG2 Cancer Cells with Respect to the Ras/Raf/MEK/ERK Pathway
Source: J Clin Med. 2026 Aug 30;15(17):6738. doi: 10.3390/jcm15176738 (PMC13566739; doi:10.3390/jcm15176738)
Supplement: Supplementary file 1 [file jcm-15-06738-s001.zip › jcm-4473155-supplementary.pdf]

Supplementary Material

Table S1. Primary and secondary antibodies and isotype controls used for flow cytometry (FACS), immunohistochemistry (IHC), and Western blot (WB).

Antibody name	Antibody type	Method	Cat no.	Manufacturer
Mouse anti-human CD73	Primary	FACS	550257	Beckman Coulter, Brea, California, USA
Mouse anti-human CD90			561558	
Mouse anti-human CD105			562408	
Mouse anti-human CD133			FAB11331P	R&D Systems, Minneapolis, Minnesota, USA
Mouse IgG1	Isotype control		555749	Beckman Coulter, Brea, California, USA
Mouse IgG1			557872	
Mouse IgG1			555751	
Mouse IgG2a			IC003P	R&D Systems, Minneapolis, Minnesota, USA
Mouse anti-human cyclin D	Primary	IHC	60186-1-Ig	Proteintech, Rosemont, Illinois, USA
Mouse anti-human alpha-fetoprotein			ab3980	Abcam, Cambridge, UK
Mouse IgG1	Isotype control		ab170190	
Goat anti-mouse	Secondary		ab175473	
Goat anti-mouse			ab150133	
Rabbit anti-human GAPDH	Primary	WB	G9545	Sigma Aldrich, Saint Louis, Missouri, USA
Mouse anti-human cyclin E			sc-247	Santa Cruz, Dallas, Texas, USA
Rabbit anti-human Hsp90			ab203085	Abcam, Cambridge, UK
Rabbit anti-human cyclin D1			ab134175	
Rabbit anti-human Ras			ab52939	
Rabbit anti-human Raf-1			ab181115	
Rabbit anti-human p-Raf-1			ab173539	
Rabbit anti-human MEK1/2			ab178876	
Rabbit anti-human p-MEK1			ab96379	
Rabbit anti-human ERK1/2			ab184699	
Rabbit anti-human p-ERK1/2			ab76299	
Rabbit anti-human cleaved caspase-7			ab256469	
Rabbit anti-Bax			50599-2-Ig	Proteintech, Rosemond, Illinois, USA

Rabbit anti-human caspase-7			27155-1-AP	
Rabbit anti-human caspase-9			10380-1-AP	
Rabbit anti-human cleaved caspase-3			9661	Cell Signaling, Danvers, Massachusetts, USA
Goat anti-rabbit	Secondary		ab205718	Abcam, Cambridge, UK
Goat anti-mouse			ab205719	

Table S2. Primers for Ras/Raf/MEK/ERK pathway and cell cycle-related genes used in RT-qPCR.

**Gene**	**Encoded protein**	**Oligo sequence (5′-3′)**	**Tm (°C)**	**GenBank transcript accession number**
HRAS	GTPase HRas	F: ACCATTTTGTGGACGAATAC<br>R: AAGACTTGGTGTTGTTGATG	87.0	NM_001318054.2
KRAS	GTPase KRas	F: GCCTGCTGAAAATGACTG<br>R: TCCTGTAGGAATCCTCTATTG	80.8	NM_001369786.1
RAF1	RAF proto-oncogene serine/threonine-protein kinase	F: ACAGATATTCTACACCTCACG<br>R: AATTGCATCCTCAATCATCC	84.7	NM_001354691.3
MAP2K1	Mitogen-activated protein kinase kinase 1	F: GATTACATAGTCAACGAGCC<br>R: CTTCAAATCTGCTCTCTCTG	79.9	NM_001411065.1
CCNE1	Cyclin E1	F: AGACATACTTAAGGGATCAGC<br>R: CACACCTCCATTAACCAATC	80.4	NM_001440307.1
CCND1	Cyclin D1	F: GTGCCACAGATGTGAAG<br>R: CTTCGATCTGCTCCTGG	80.2	NM_053056.3
ACTB	Actin beta	F: GACGACATGGAGAAAATCTG<br>R: ATGATCTGGGTCATCTTCTC	89.0	NM_001101.5

Table S3. Primers for *AFP* and apoptosis-related genes used in RT-qPCR.

**Gene**	**Encoded protein**	**Oligo sequence (5′-3′)**	**Tm (°C)**	**GenBank transcript number**
AFP	Alpha fetoprotein	F: GATCCCACTTTTCCAAGTTC<br>R: TTTGTTCATGAATGTCTCCC	80.2	NM_001134.3
BAX	Apoptosis regulator BAX	F: AACTGGACAGTAACATGGAG<br>R: TTGCTGGCAAAGTAGAAAAG	86.5	NM_138764.5
CASP3	Caspase 3	F: AAAGCACTGGAATGACATC<br>R: CGCATCAATTCCACAATTTC	80.5	NM_001354782.2
CASP7	Caspase 7	F: AAGCCATGGAGAAGAAAATG<br>R: CCTGAATGAAGAAGAGTTTGG	80.1	NM_001267057.1
TP53	Tumor protein p53	F: ACCTATGGAAACTACTTCCTG<br>R: ACCATTGTTCAATATCGTCC	82	NM_001276761.3
ACTB	Actin beta	F: GACGACATGGAGAAAATCTG<br>R: ATGATCTGGGTCATCTTCTC	89	NM_001101.5

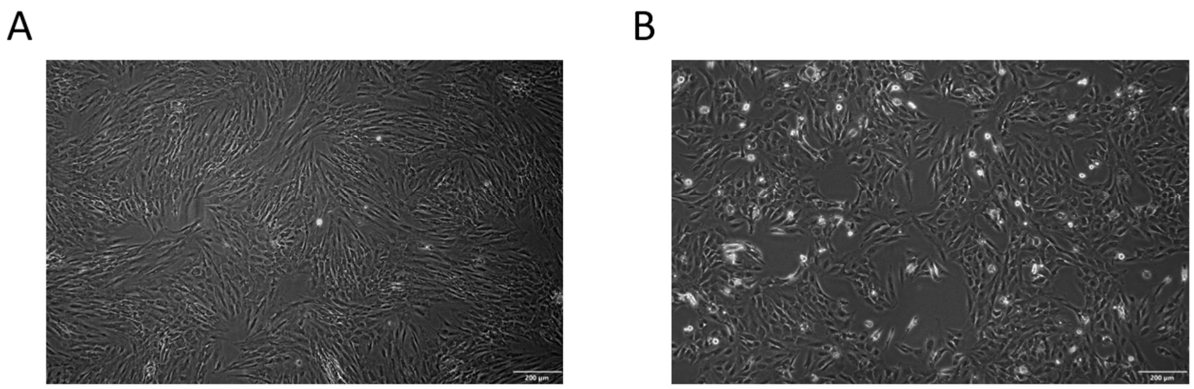

Figure S1. Cell cultures in standard medium. **(A)** Spindle-shaped human adipose tissue-derived mesenchymal stem cells (hADSCs). **(B)** Human amnion derived cells (hACs) consisting of spindle-shaped and cuboidal cells. Scale bar = 200 μm.

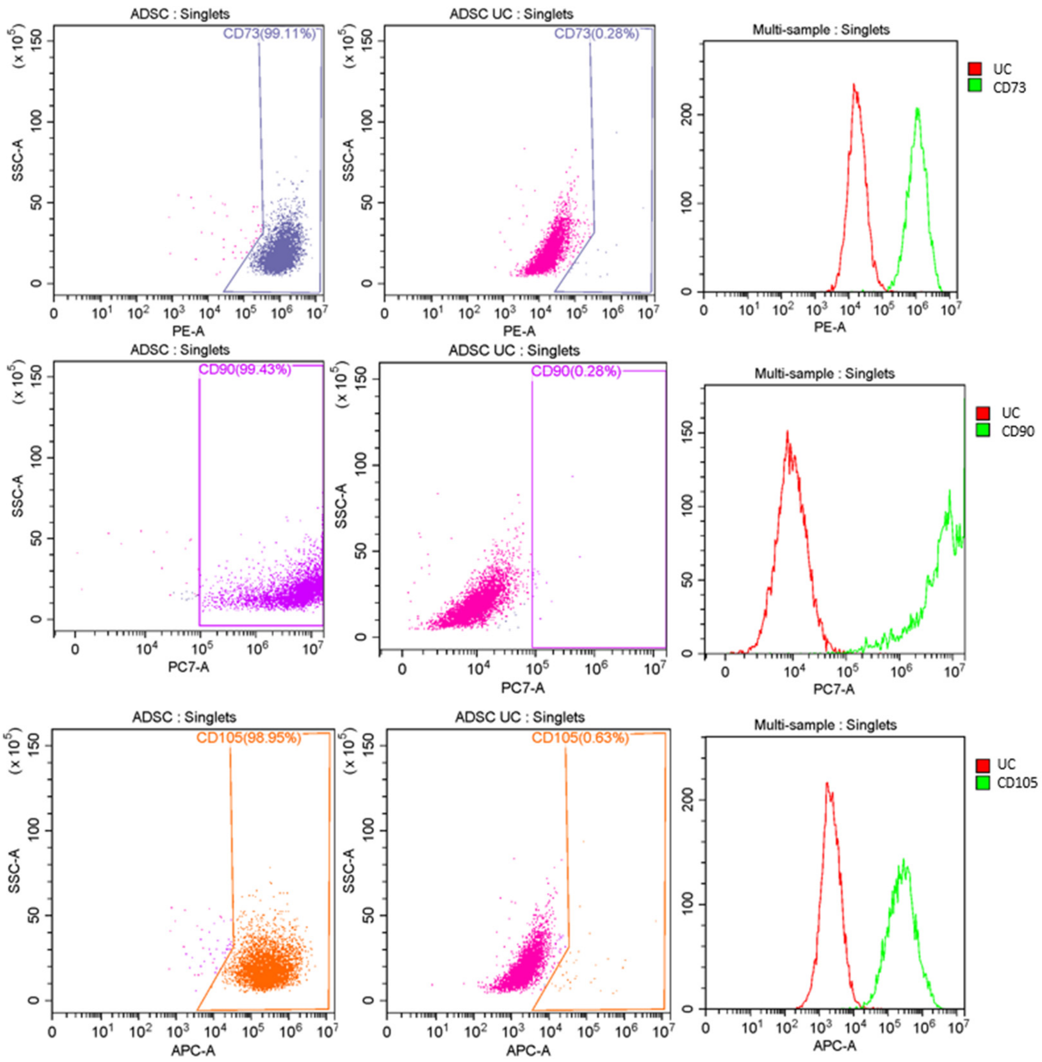

Figure S2. Expression of mesenchymal surface markers on hADSCs (passage 3). The percentage of cells expressing individual markers is given. UC – unstained control.

A

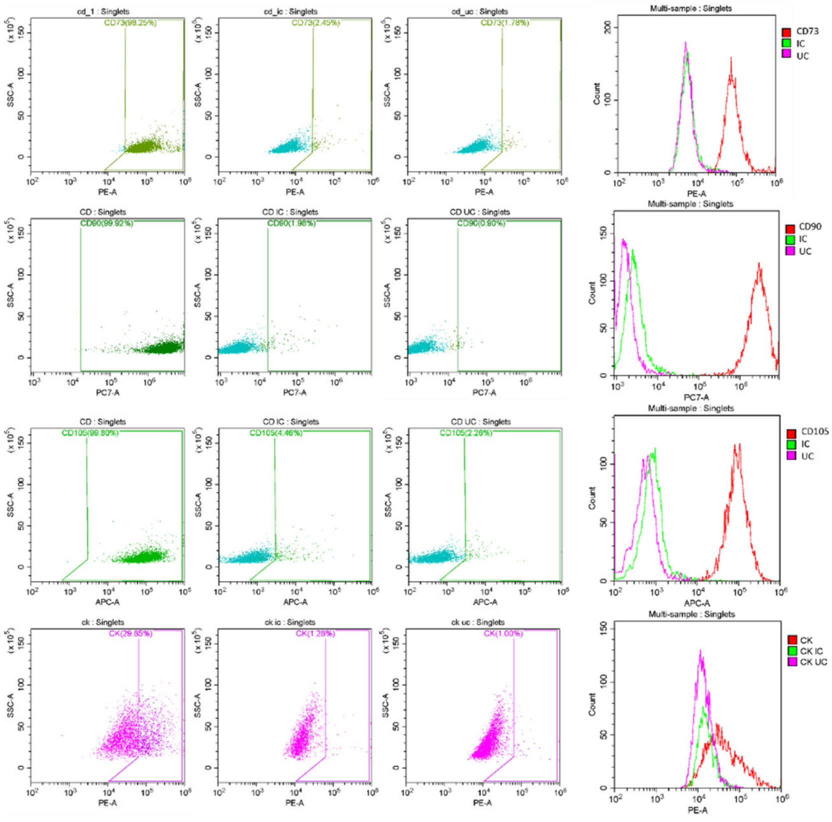

B

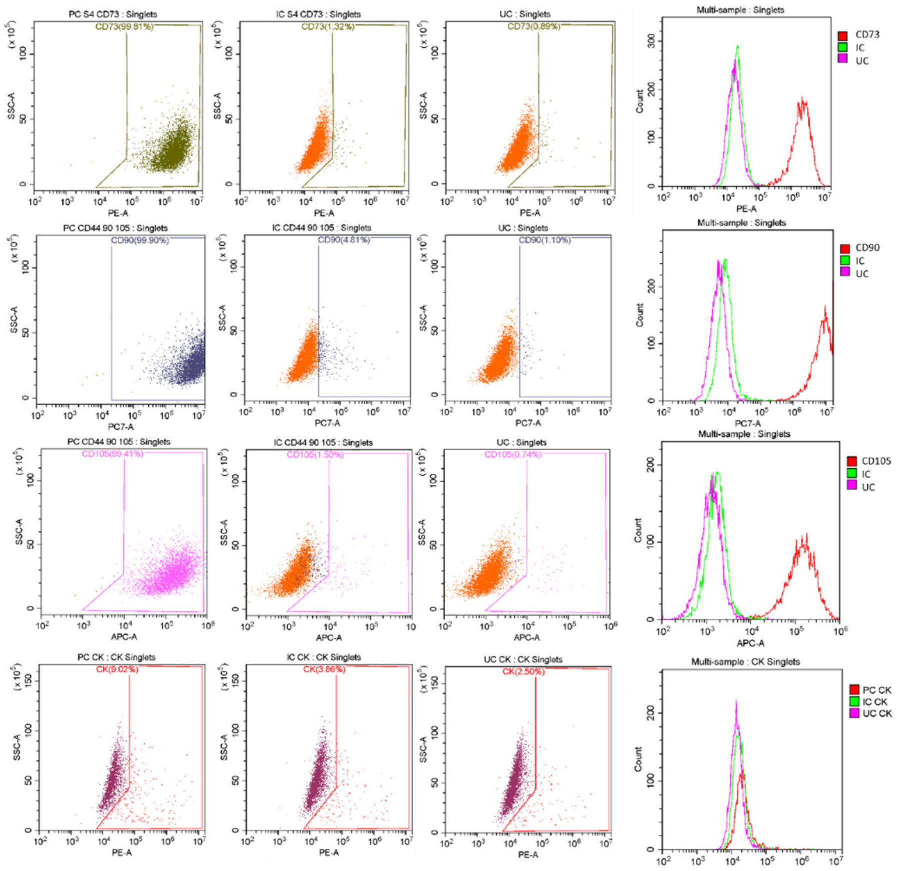

Figure S3. Expression of epithelial and mesenchymal surface markers in hACs. **A** – passage 2, **B** – passage 7. The percentage of cells expressing individual markers is given; CK – cytokeratins; IC – isotype control; UC – unstained control.

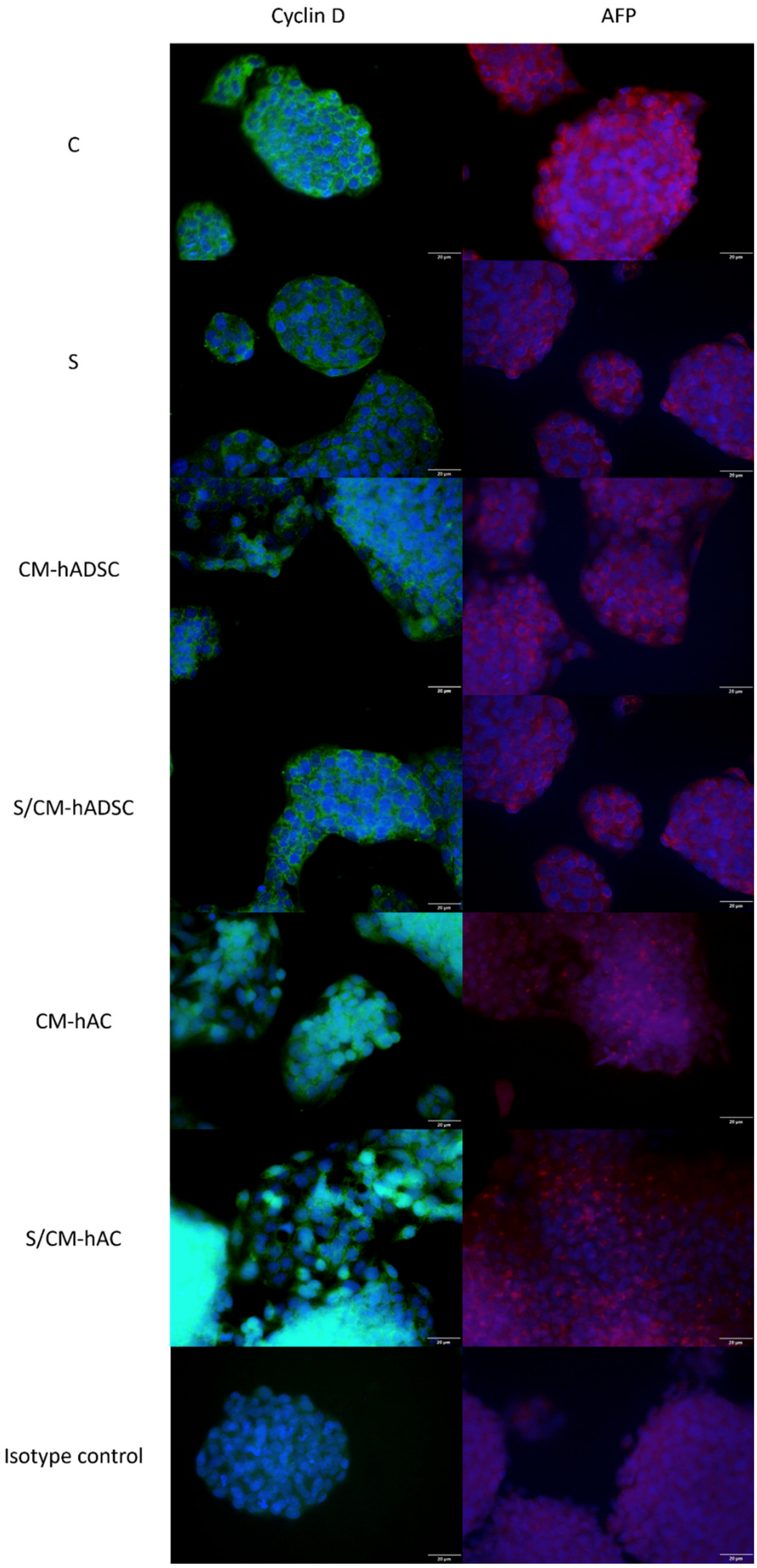

Figure S4. Immunodetection of cyclin D (left) and AFP (right) proteins in HepG2 cells treated with sorafenib and/or CM-hADSC and CM-hAC. Nuclei were stained with DAPI. Scale bar = 20 μm.

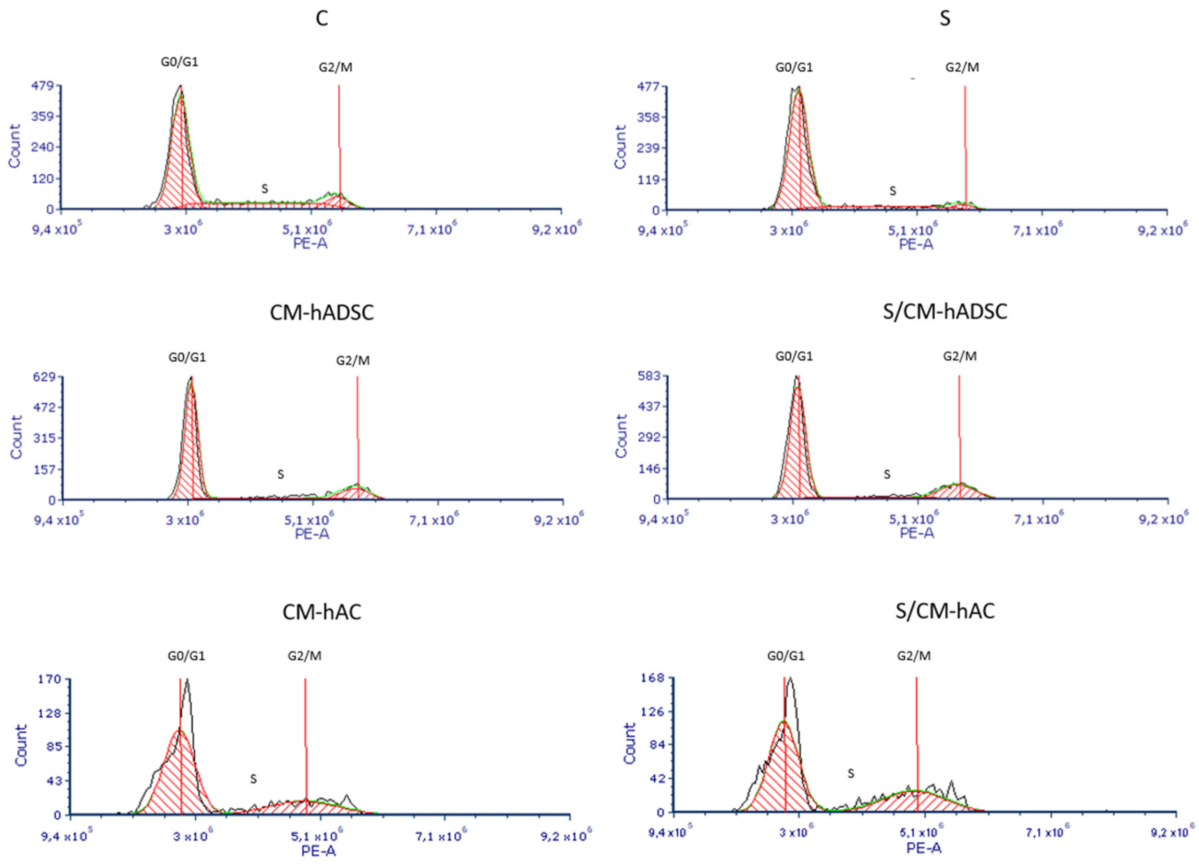

Figure S5. Comparison of cell cycle phase distribution in HepG2 cells after treatment with sorafenib and/or CM. G1/G0 – first peak; G2/M – second peak; S – between the first and second peaks.

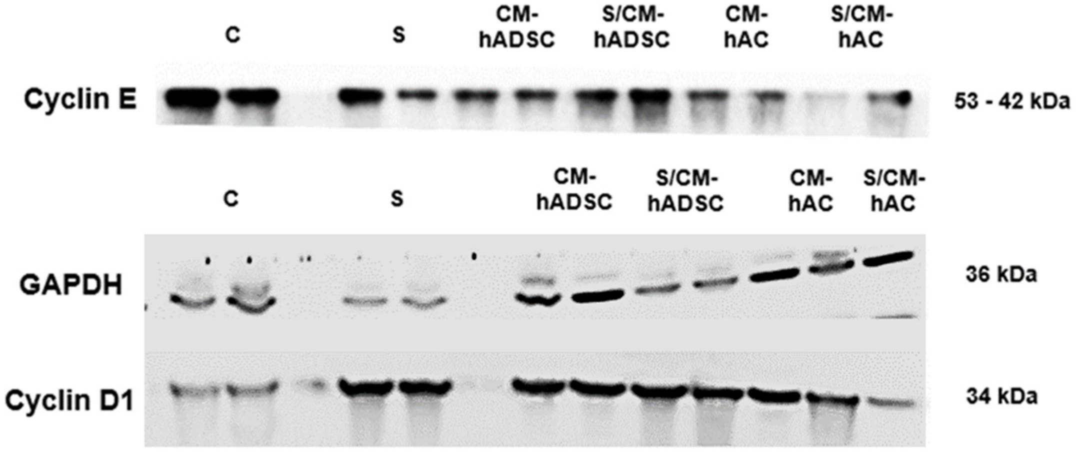

Figure S6. Representative visualization (Western blot) of cyclin E and cyclin D1 proteins in HepG2 cells after culture with sorafenib and/or hADSC- or hAC-derived CM.
